# Feeding with Sustainably Sourdough Bread Has the Potential to Promote the Healthy Microbiota Metabolism at the Colon Level

**DOI:** 10.1128/Spectrum.00494-21

**Published:** 2021-12-01

**Authors:** Alessio Da Ros, Andrea Polo, Carlo Giuseppe Rizzello, Marta Acin-Albiac, Marco Montemurro, Raffaella Di Cagno, Marco Gobbetti

**Affiliations:** a Faculty of Science and Technology, Libera Universitá di Bolzano, Bolzano, Italy; b Department of Environmental Biology, “Sapienza” University of Rome, Rome, Italy; c Department of Soil, Plant and Food Science, University of Bari Aldo Moro, Bari, Italy; University of Melbourne

**Keywords:** SCFA, SHIME, gut microbiota, metabolome, sourdough

## Abstract

The contribution of sustainably food processing to healthy intestinal microbial functions is of recent acquisition. The sourdough fermentation fits well with the most sustainable bread making. We manufactured baker’s yeast (BYB) and sourdough (t-SB30) breads, which first underwent to an in-depth characterization. According to nutritional questionnaires, we selected 40 volunteers adhering to the Mediterranean diet. Data on their fecal microbiota and metabolome allowed the selection of two highly representative fecal donors to separately run the Twin Mucosal-SHIME (Twin M-SHIME) under 2-week feeding with BYB and t-SB30. Bread feeding did not affect the microbial composition at phylum and family levels of both donors, in all Twin M-SHIME colon tracts, and lumen and mucosal compartments. The genus core microbiota showed few significant fluctuations, which regarded the relative abundances of Lactobacillus and Leuconostoc according to feeding with BYB and t-SB30, respectively. Compared with BYB, the content of all short chain fatty acids (SCFA), and isovaleric and 2-methylbutyric acids significantly increased with t-SB30 feeding. This was evident for all Twin M-SHIME colon tracts and both donors. The same was found for the content of Asp, Thr, Glu, GABA, and Orn. The bread characterization made possible to identify the main features responsible for this metabolic response. Compared with BYB, t-SB30 had much higher contents of resistant starch, peptides, and free amino acids, and an inhomogeneous microstructure. We used the most efficient approach to investigate a staple food component, excluding interferences from other dietary factors and attenuating human physiology overlaps. The daily consumption of sourdough bread may promote the healthy microbiota metabolism at colon level.

**IMPORTANCE** Knowledge on environmental factors, which may compose the gut microbiota, and drive the host physiology and health is of paramount importance. Human dietary habits and food compositions are pivotal drivers to assemble the human gut microbiota, but, inevitably, unmapped for many diet components, which are poorly investigated individually. Approximately 30% of the human diet consists of fermented foods and beverages. Bread, a fermented/leavened food, is a basic component of the human diet. Its potential effect on gut microbiota composition and functionality is challenging. In this study, we industrially made baker’s yeast and sourdough breads, which were used to feed the Twin Mucosal-SHIME, a worldwide scientifically validated gastrointestinal simulator. Only the consumption of sourdough bread has the potential to enhance the synthesis of short chain fatty acids and free amino acids at the colon level.

## INTRODUCTION

The human gastrointestinal tract is a highly complex system, where multi-dynamic physiological processes are tightly coordinated via interaction with an abundant and diverse microbiota (1). The challenge is to keep this mutualistic relationship throughout life, which relies on gut microbiota composition and functionality. Nowadays, we have the confirmation that components of the gut microbiota prevent entero-pathogens, provide energy and essential nutrients, improve nutrient digestibility, maintain optimal gut motility and immune system activity, and, probably, release chemicals involved in cancer prevention (2). Conversely, the intestinal dysbiosis aggravates various diseases, like obesity, Type 2 diabetes, and inflammatory bowel syndrome (IBS) (3). These concepts strengthen the need to identify environmental factors, which may compose the gut microbiota and drive the host physiology and health.

Since our birth, diet is a pivotal driver to assemble the human gut microbiota by providing specific nutrients and inducing environmental changes (e.g., pH, bile acids) (2). The descriptions of human dietary habits and food compositions are challenging and, inevitably, unmapped for many diet components, which are poorly investigated individually (4). Approximately 30% of the human diet consists of fermented foods and beverages (5). Although the fermentation was historically used to extend shelf life, nowa days fermented foods have gained popularity because of their health-promoting benefits (6), including their potential relevance for the gut microbiota (7). Bread is a basic component of the human diet, with an estimated per capita daily consumption of 180–250 g (8). Roughly, bread counts for ca. 10% of the adult caloric intake (9). Bread at industrial and artisanal levels is mostly made by fermentation with baker’s yeast or sourdough fermentations. Sourdough is a mixture of flour and water, spontaneously fermented by lactic acid bacteria and yeasts, and having acidification and leavening capacities (10). Dominant and subdominant lactic acid bacteria and yeasts come spontaneously from flours, mainly as plant endophytes, and the house microbiota (11). Because of this inevitable microbial diversity and its repercussion on the nutritional potential, the sourdough fermentation was deeply investigated for its effects on mineral bioavailability, glycemic index, digestibility, and the capability to decrease the content of antinutritional compounds (11). Its potential effect on gut microbiota composition and functionality represents the new challenge. Available reports have mainly dealt with the *in vitro* or *in vivo* effects of sourdough breads on individuals suffering from celiac disease and IBS. Attenuation of the disease symptoms or decreased levels of FODMAP and gluten were demonstrated (12, 13). Two other studies using fecal slurries or rats (14, 15) dealt with the effect of processing methods on microbiota composition and functionality. Only one *in vivo* randomized crossover study (16) focused on the comparison between traditionally made sourdough whole-grain bread and industrially made white bread. The sourdoughs used in almost all of these studies did not come from a previous selection, lacked specifications about the microbiota composition, and, more in general, the *in vivo* approach made it difficult to extrapolate data only attributable to bread consumption because of the inevitable interindividual differences on dietary habits.

Human feces are the preferred choice for analyzing the gut microbiota because intestinal biopsies are difficult or impossible to obtain (2). *In vivo* studies also relied on data from fecal samples, which have the limitations to not describe the gut microbiota composition and activities from colon regions, and to not differentiate from lumen and mucosa (17). Furthermore, the strong interplay between host cells and resident microbes and their metabolites makes it difficult to distinguish between their contributions. Thus, *in vitro* research using gastrointestinal simulators became an essential tool especially to map the effect of individual diet components. SHIME is the acronym for the Simulator of the Human Intestinal Microbial Ecosystem, which, after the first publication in 1993 (18), had many validations (148 scientific articles retrieved by Scopus, May 2021) and became a dynamic multicompartmental model capable of reproducing a stable human gut microbial community for nutritional and pharmaceutical studies.

In this study, we industrially made baker’s yeast and selected sourdough breads, which, after a preliminary biochemical, textural, and nutritional characterization, were used to feed the Twin Mucosal-SHIME (Twin M-SHIME) separately inoculated with feces from two donors representative of healthy individuals adhering to the Mediterranean diet (MD). Metagenomics and metabolomics were used to estimate the differential effects of the two types of bread on gut microbiota composition and functionality.

## RESULTS

### Manufacture of traditional sourdough and baker`s yeast breads.

Type-I sourdough (S) was made using selected strains of sourdough lactic acid bacteria and yeasts, which ensured bread making performance and reproducibility (19). S became mature after four daily refreshments (Table S1) and it was used to prepare the sourdough for bread making with an incubation for 24 h at 30°C (S_24_). S_24_ had cell densities of lactic acid bacteria and yeasts of 9.8 and 7.5 log CFU/g, respectively. A relevant production of lactic and acetic acids was observed, with resulting fermentation quotient (FQ) of 4.4. S_24_ had a content of free amino acids (FAA) significantly (*P  < * 0.05) higher than S. Bread made with 30% (wt/wt) of S_24_ (t-SB30) or baker’s yeast bread (BYB) were manufactured (Fig. S1). The proximal composition of the two breads (Table S2) did not show (*P  > * 0.05) significant differences in macronutrients. As expected, t-SB30 had the lowest (*P  < * 0.05) value of pH value (4.2 ± 0.1) due to lactic acid fermentation (Table 1). Compared with BYB, t-SB30 had much higher contents of peptides (9.3 ± 0.1 g/kg) and FAA (2.16 ± 0.06 g/kg). Also, most of the individual FAA were at the highest levels in t-SB30 (Fig. S2), mainly Leu and Glu, followed by Ile, Val, and Asp. Compared with BYB, the volume of t-SB30 was slightly but significantly (*P  < * 0.05) lower. The specific volume agreed and the value of hardness of t-SB30 was significantly (*P  < * 0.05) higher. Contrarily, t-SB30 showed the lowest fracturability, which corresponded to a remarkable friability and resilience of the crumb. This last attribute was an index of elasticity. All biochemical and textural data proved that we manufactured traditional sourdough and baker’s yeast breads (19).

**TABLE 1:** Bread chemical, technological, and nutritional characteristics. BYB, baker’s yeast bread made mixing wheat flour (62.5%, wt/wt), water (37.5%, wt/wt), and 1.5% (wt/wt) of baker’s yeast and fermented for 2 h at 30°C; t-SB30, sourdough bread made mixing 30% (wt/wt) of sourdough S_24_ (fermented for 24 h at 30°C, step I) with flour (46.7% wt/wt) and water (23.3% wt/wt) and fermented for 4 h at 30°C (step II)

Chemical, technological, and nutritional characteristics	BYB	t-SB30
Dough (before baking)		
pH	5.6 ± 0.1^*a*^	4.2 ± 0.1^*b*^
TTA (mL NaOH 0.1 m/10 g)	2.9 ± 0.1^*b*^	8.5 ± 0.2^*a*^
Lactic acid (mmol/kg)	1.2 ± 0.1^*b*^	32.2 ± 0.4^*a*^
Acetic acid (mmol/kg)	Nd	7.7 ± 0.1
FQ	Nd	4.2 ± 0.2
Total FAA (g/kg)	0.61 ± 0.06^*b*^	2.16 ± 0.06^*a*^
Bread		
Vol increase (%)	245 ± 5^a^	210 ± 4^*b*^
Specific vol (cm^2^/g)	3.3 ± 0.1^a^	2.7 ± 0.1^*b*^
* Textural parameters*		
Hardness (g)	3230 ± 22^*b*^	3494 ± 18^*a*^
Resilience	0.85 ± 0.02^*a*^	0.69 ± 0.02^*b*^
Fracturability (g)	3080 ± 5^*a*^	2168 ± 10^*b*^
* Image analysis*		
Black pixel area (%)	48.0 ± 1.8^*b*^	41.1 ± 1.3^*b*^
* Nutritional indexes*		
*In vitro* protein digestibility (IVPD, %)	64.2 ± 0.7^*b*^	82.6 ± 1.5^*a*^
Total peptides (g/kg)	4.4 ± 0.1^*b*^	9.3 ± 0.1^*a*^
	Lysine	Lysine
Limiting amino acids	Methionine	Methionine
	Tryptophan	Tryptophan
Protein score (%)	18.5 ± 0.3^*b*^	62.0 ± 0.5^*a*^
Essential amino acids index (EAAI)	43.0 ± 1.0^*b*^	73.5 ± 0.6^*a*^
Biological value (BV)	36.5 ± 0.5^*b*^	68.4 ± 1.2^*a*^
Protein efficiency ratio (PER)	22.5 ± 0.7^*b*^	54.6 ± 0.4^*a*^
Nutritional index (NI)	2.5 ± 0.4^*b*^	5.8 ± 0.2^*a*^
Predicted glycemic index (pGI)	95^*a*^	72^*b*^
Resistant starch (%, d.m.)	1.1 ± 0.1^*b*^	1.8 ± 0.1^*a*^

a,b Values in the same row with different superscript letters differ significantly (*P  *< * *0.05) based on one-way ANOVA (Tuckey-Kramer). The data are the means of three independent analysis ± standard deviations (*n* = 3).

### Baker’s yeast and sourdough breads had distinguishing nutritional indexes.

The value of *in vitro* protein digestibility (IVPD) of BYB was ca. 64.2% (Table 1). The sourdough fermentation significantly (*P  <  *0.05) increased the value of IVPD to ca. 82.6%. Lys, Met, and Trp were the limiting amino acids for both breads. The protein score was significantly (*P  < * 0.05) higher in t-SB30. Similarly, the values of essential amino acid index (EAAI), biological value (BV), and protein efficiency ratio (PER), which estimated the food protein quality, were significantly (*P  <  *0.05) higher in t-SB30. The nutritional index (NI), whose calculation refers to both the amount of digestible protein fraction and the ratio of essential amino acids, also agreed. According to the starch hydrolysis index (HI) (58.8), the predicted glycemic index (pGI) of t-SB30 was ca. 72, markedly lower (*P  <  *0.05) than that of BYB. Compared with BYB, t-SB30 had a higher content of resistant starch (1.79 ± 0.04 versus 1.08 ± 0.05%, d.m.). The difference in resistant starch was amplified when the analyses were repeated sampling the lumen compartment of the Twin M-SHIME colon tracts after bread feeding (2.36 ± 0.19 versus 3.21 ± 0.15 g/l for BYB and t-SB30, respectively).

### Baker’s yeast and sourdough breads had distinguishing microstructure.

As observed by confocal laser scanning microscope (CLSM) (Fig. S3), discontinuous protein-rich areas with the dominant presence of starch granules were observed in t-SB30. This demonstrated a structural inhomogeneity. BYB showed a more interconnected network of gluten strands surrounding starch particles.

### Fecal donors representatives of high adherence to Mediterranean diet.

According to nutritional questionnaires, 40 of the recruited volunteers satisfactorily adhered to MD. Their averaged MD score (MDS) was 5 ± 1. The aggregate microbiota composition of their fecal samples showed the dominance of Firmicutes (77.7%) and Bacteroidetes (6.9%), followed by Actinobacteria, Proteobacteria, and other minor phyla (Fig. S4). The ratio between Firmicutes and Bacteroidetes was 14.4. The concentration of short chain fatty acids (SCFA) and their derivatives (isobutyric, isovaleric, 2-methylbutyric, valeric, hexanoic, and hexadecenoic acids) agreed with the features of individuals adhering to MD (20). Although with interindividual variability, the cohort of 40 individuals showed the overall dominance of Lachnospiraceae, followed by Bacteroidaceae and Clostridiaceae families (Figure S5a). Other families showing variable abundances were Coriobacteriaceae, Streptococcaceae, and Vellionellaceae. Data on microbiota composition and SCFA and their derivatives were statistically elaborated. A preliminary heatmap analysis grouped the 40 individuals into three clusters (Fig. S6). Validation test through partial least square discriminant analysis (PLS-DA) confirmed the statistical significance of the clustering, where components 1 and 2 explained 77.87% and 0.05% of the total variance (Figure S5b). Individuals with high adherence to MD (MDS score > 5) formed two clusters, while those with satisfactory but lower adherence (MDS score between 4 and 5) grouped into a separate cluster. The PLS-DA model also described the contribution of each variable to the two loading components, and highlighted the bacterial taxa and SCFA that clustered the individuals with high MD adherence into two groups (Figure S5c). The relative abundance of Ruminococcaceae, Clostridiaceae, Streptococcaceae, Bacteroidaceae, and Lachnospiraceae and butyric acid mainly determined the aggregation of group 1 (High-AD_G1), while Enterococcaceae and hexadecanoic acid mainly affected group 2 (High-AD_G2). Based on these results, we excluded the four individuals falling into group 3 (satisfactory but lower adherence to MD) and randomly selected the fecal donors SB11 and SB33 as representatives for groups 1 and 2, respectively.

### Feeding with baker’s yeast and sourdough breads only slightly affected the core microbiota.

**FIG 1:**
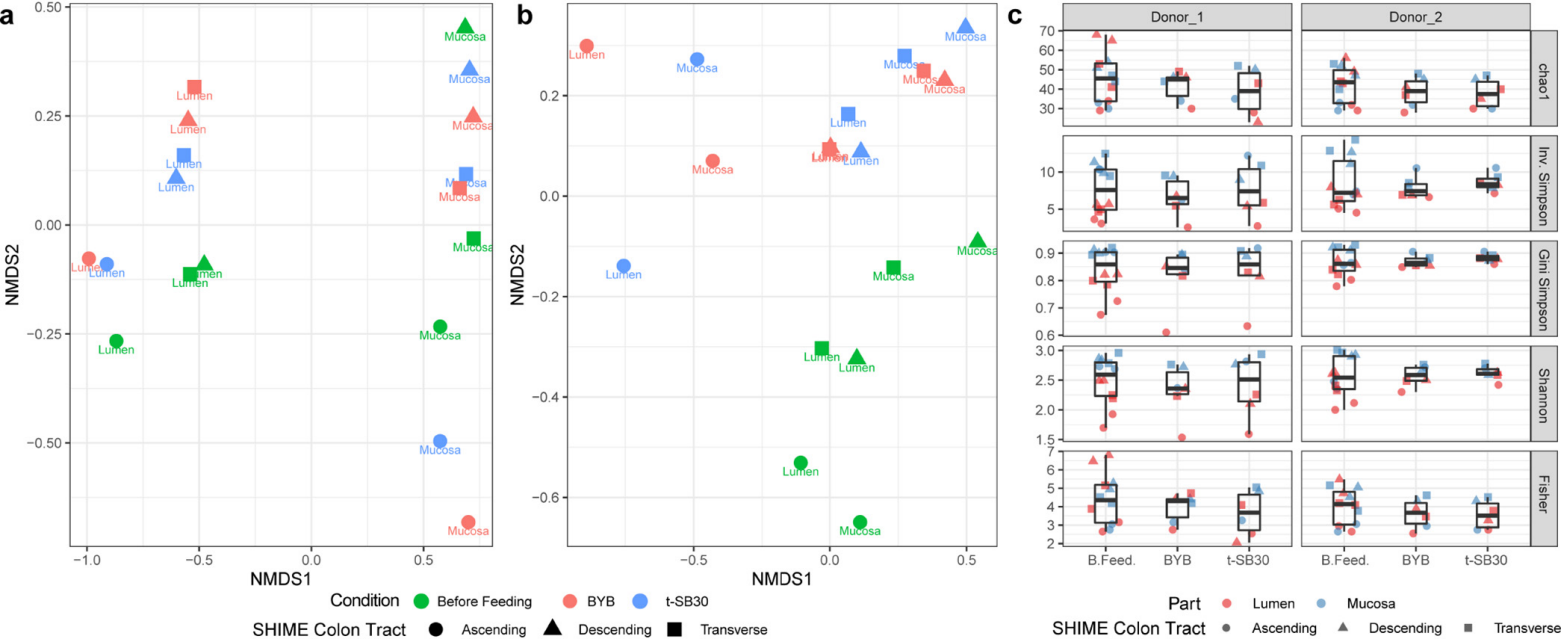
Nonmetric multidimensional scaling (NMDS) analysis of the microbiota based on Bray-Curtis distance matrix of donor 1 (a) and donor 2 (b). The analysis considered sample interdiversity of Twin M-SHIME colon tracts (ascending, transverse, and descending), lumen and mucosal compartments, and before and after 14 days of feeding with sourdough (t-SB30) and baker’s yeast (BYB) breads. Panel (c) shows the total distribution of alpha diversity determined with chao1, inverse Simpson, Gini Simpson, Shannon, and Fisher indexes of luminal and mucosal microbiota in the three colon tracts before (B.Feed.) and after feeding with t-SB30 and BYB breads.

**FIG 2:**
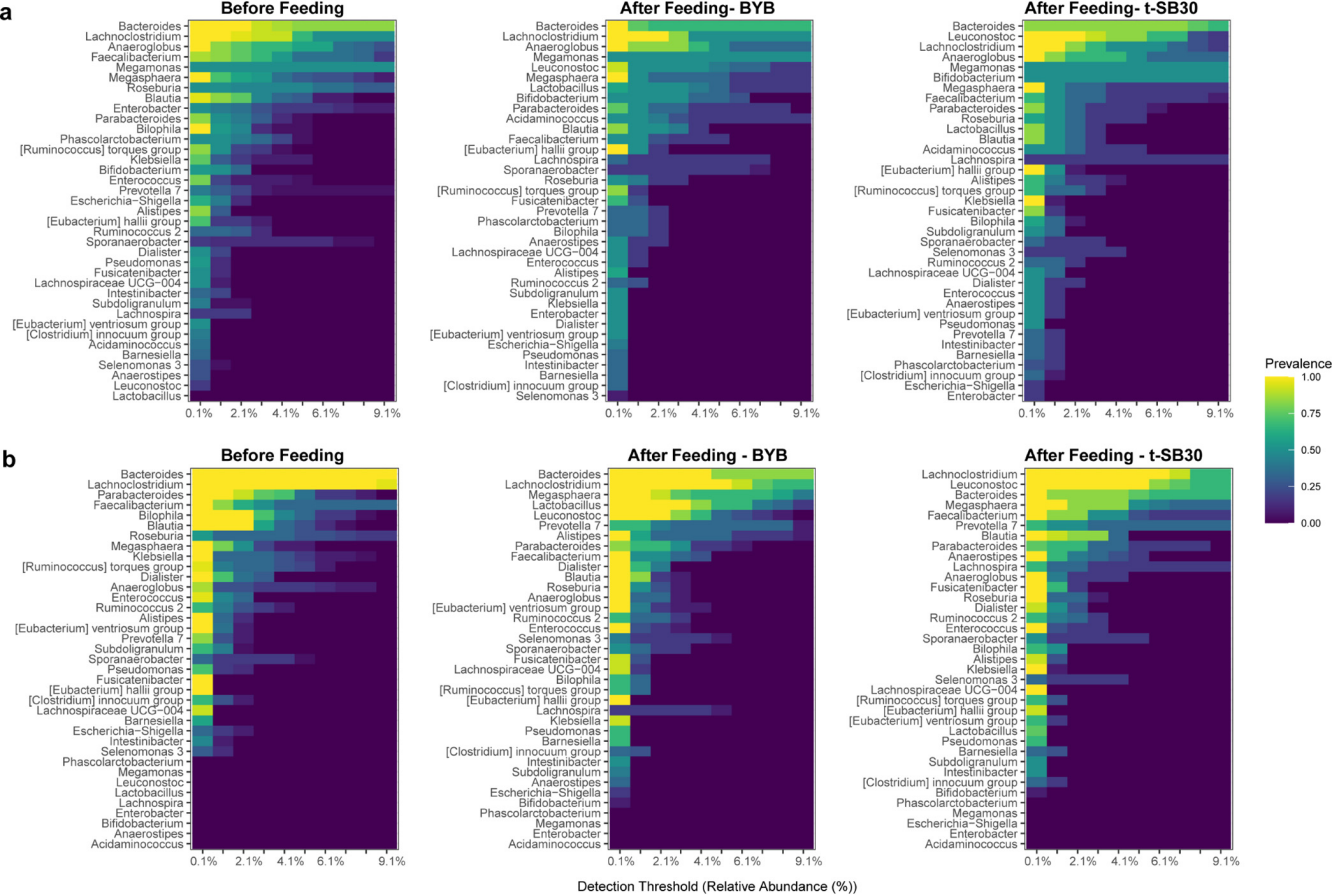
Pseudo-heatmaps showing the relative abundances of the genus core microbiota of all Twin M-SHIME colon tracts (lumen and mucosal compartments) before and after feed- ing with baker’s yeast (BYB) and sourdough (t-SB30) breads. Panels (a) and (b) refer to donors 1 and 2, respectively. The color scale indicates the prevalence of OTU abundances across different detection thresholds ranging from 0.1% to 9.1%.

**FIG 3:**
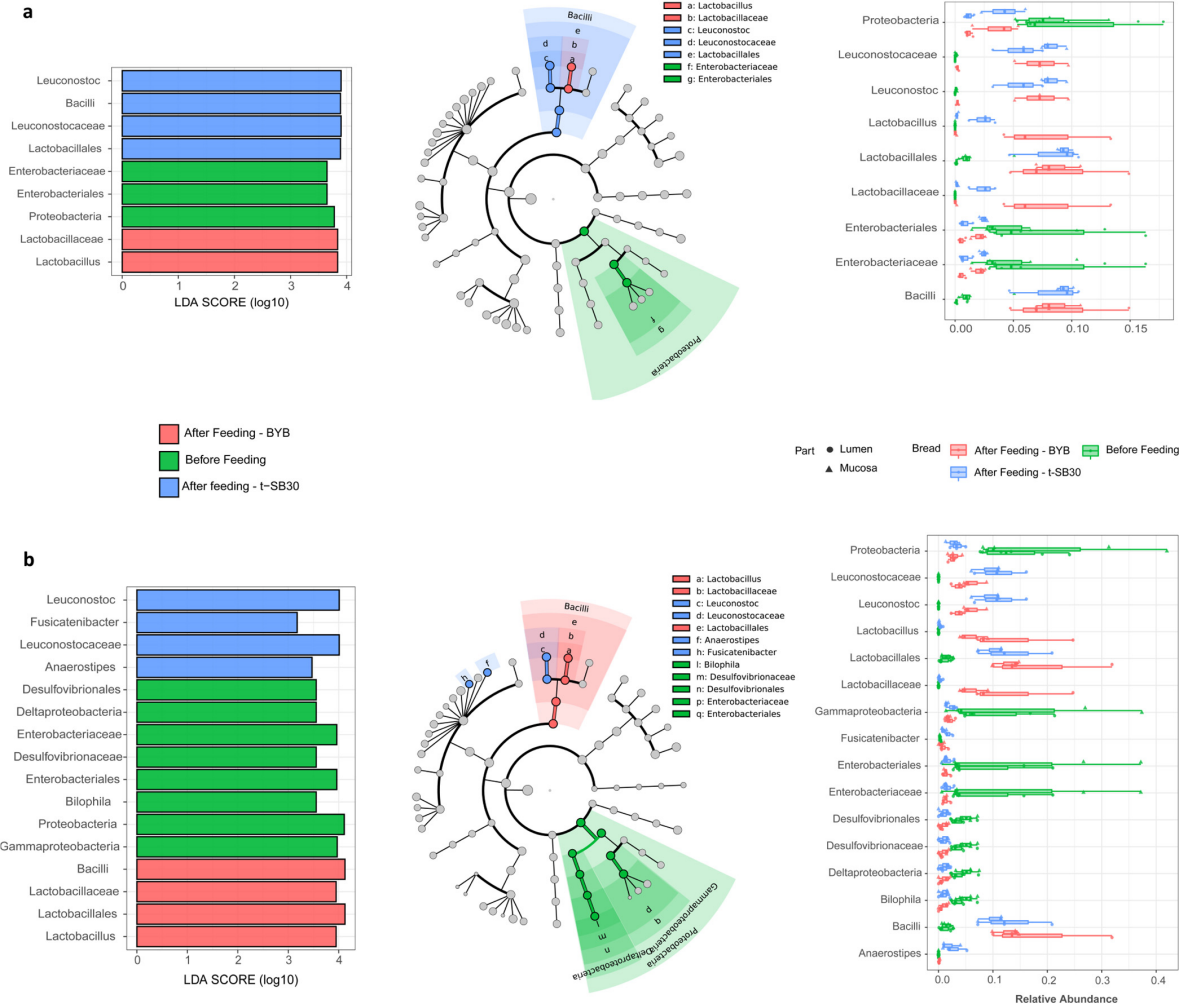
Linear discriminant analysis (LDA) of the microbiome at different taxonomic levels (left) and boxplot showing relative abundances across SHIME tracts and parts (right) before and after the feeding with baker’s yeast (BYB) and sourdough (t-SB30) breads. Panels (a) and (b) refer to donors 1 and 2, respectively. Colors, green refers to the genus core microbiota before bread feeding, and blue and red refer to the genus core microbiota after administration of t-SB30 and BYB, respectively.

Two runs, separately using one of the two selected fecal donors, were carried out with the Twin M-SHIME. The sampling of lumen and mucosal compartments was done for each of the Twin M-SHIME colon tracts. Each run compared BYB versus t-SB30 breads. Although sampling was three times per week, the results almost exclusively referred to data at the end of bread feeding because the variations over time always demonstrated the same trend (Fig. S7). After stabilization and steady state (4 weeks) of the Twin M-SHIME (before bread feeding), the relative abundances of microbial phyla for both donors and all Twin M-SHIME colon tracts (lumen and mucosal compartments) were consistent with those observed initially in the fecal materials. Similarities were also found among colon tracts of both donors, except for Actinobacteria, which was detected only in the lumen compartments of donor 1 (Figure S8a). Overall, Firmicutes and Bacteroidetes, followed by Proteobacteria, were the taxa with the highest relative abundances both in lumen and in mucosal compartments of both donors and all Twin M-SHIME colon tracts. The phylum distribution of both donors only slightly varied after 14 days of feeding with both t-SB30 and BYB breads (Figure S8b and S8c, respectively). The only changes were an increase of the relative abundance of Firmicutes in the ascending colon of both donors (lumen and mucosal compartments), and the decrease of Proteobacteria and Bacteroidetes. Actinobacteria increased in the lumen compartment of all Twin M-SHIME colon tracts of donor 1. These variations depended on bread feeding regardless of the type of bread. Also, at the family level (Fig. S9), the relative abundances of Twin M-SHIME colon tracts of both donors at the steady state mirrored the microbiota composition of the fecal materials. Lachnospiraceae and Bacteroidaceae were the most abundant families of both aggregate microbiota. In detail, the lumen compartment of all Twin M-SHIME colon tracts of donor 1 had higher abundance of Veillonellaceae and Acidaminococcaceae and lower abundances of Lachnospiraceae and Bacteroidaceae with respect to donor 2. Differences between the two donors were less pronounced in the mucosal compartments. After bread feeding (14 days), the relative abundances of Bacteroidaceae and Enterobacteriaceae decreased in the ascending colon of both donors (lumen and mucosal compartments). For the same colon tract, feeding with BYB seemed to promote a higher relative abundance of Lactobacillaceae. Changes of relative abundances for transverse and descending colon, and both donors, were slight and regardless of the type of bread. Almost the same trend was observed after 7 days of bread feeding. Nonmetric multidimensional scaling (NMDS) analysis (Fig. 1) confirmed that feeding with t-SB30 or BYB breads did not affect the microbiota of the Twin M-SHIME colon tracts differently (lumen and mucosal compartments). We assessed various diversity indexes to estimated eventual changes, but all showed no statistical differences (*P*  >  0.05). Diversity was also assessed between the initial microbiota and that observed after 14 days of bread feeding. NMDS graphs showed a separation between the initial microbiota and those found for both donors after bread feedings, but the overall intradiversity of lumen and mucosal microbiota of all three Twin M-SHIME colon tracts did not statistically differ (*P* > 0.05) (Fig. 1). To search for some diversity, we used different detection thresholds to investigate the genus core microbiota before and after bread feeding, and we merged all Twin M-SHIME colon tracts and lumen and mucosal compartments (Fig. 2). Before feeding, *Bacteroides* and *Lachnoclostridium* were the prevalent taxa in the core microbiota of both donors, followed by *Roseburia*, *Blautia*, *Faecalibacterium*, and *Megasphera*. Although *Prevotella*, Pseudomonas, *Sporanaebacter*, Eubacterium hallii group and *Alistipes* were part of the core microbiota, their relative abundances were lower with respect to the previous genera. Also the composition of the genus core microbiota of both donors only slightly changed after bread feeding. When switching was observed, it was almost regardless of the type of bread and almost exclusively concerned a re-raking within the core microbiota (Fig. 2). After feeding with t-SB30 bread, *Leuconostoc* became one of the most prevalent taxa for both donors. The relative abundance of *Lactobacillus* behaved similarly which mainly increased after feeding with BYB bread. Regarding donor 1, the relative abundance of *Bifidobacterium* also increased mainly after feeding with t-SB30 bread. *Bacteroides* was the highest abundance within the genus core microbiota. The only exception concerned donor 2 after feeding with t-SB30 bread, which favored the overcoming by *Leuconostoc* and *Lachnoclostridium.* Despite *Lachnoclostridium* being the second most prevalent genus before bread feeding and after administration of BYB bread for both donors, t-SB30 bread had contrasting effects on its relative abundance. For donor 1, t-SB30 promoted the overcoming by *Leuconostoc*, while donor 2 favored the dominance of *Lachnoclostridium*. *Lachnospira* turned as part of the genus core microbiota only for donor 2. Although *Anaerostipses* and *Bifidobacterium* were part of the genus core microbiota of donor 1 before and after bread feeding, for donor 2 they became part of the genus core microbiota only after bread feeding. The relative abundance of *Biophila* of both donors decreased after feeding with BYB and t-SB30 breads. Escherichia-shigella similarly trended in donor 1, while the relative abundance of Ruminococcus torques group decreased only for donor 2. Linear discriminant analysis (LDA), together with related boxplots, summarized the most significant fluctuations of the genus core microbiota and their related taxonomic levels after bread feeding (Fig. 3). In agreement with Fig. S8, the Proteobacteria phylum and especially the Enterobacteriaceae family were the main microbiota drivers for both donors before bread feeding. *Leuconostoc* and *Lactobacillus* genera were the main drivers for both donors after feeding with t-SB30 and BYB breads, respectively. This agreed with genus rearrangement of the core microbiome (Fig. 2). Within the Lachnospiraceae family, *Anaerostipes* genus was the main driver for donor 2 after feeding with t-SB30. At higher taxonomic level (Bacilli), bread feeding had a donor dependent effect. Following 1 week of wash out, the microbiota of all Twin M-SHIME colon tracts (lumen and mucosal compartments) of both donors trended to similar relative abundances as those observed before bread feeding.

### Sourdough bread enhanced the synthesis of SCFA and γ-amino butyric acid (GABA).

**FIG 4:**
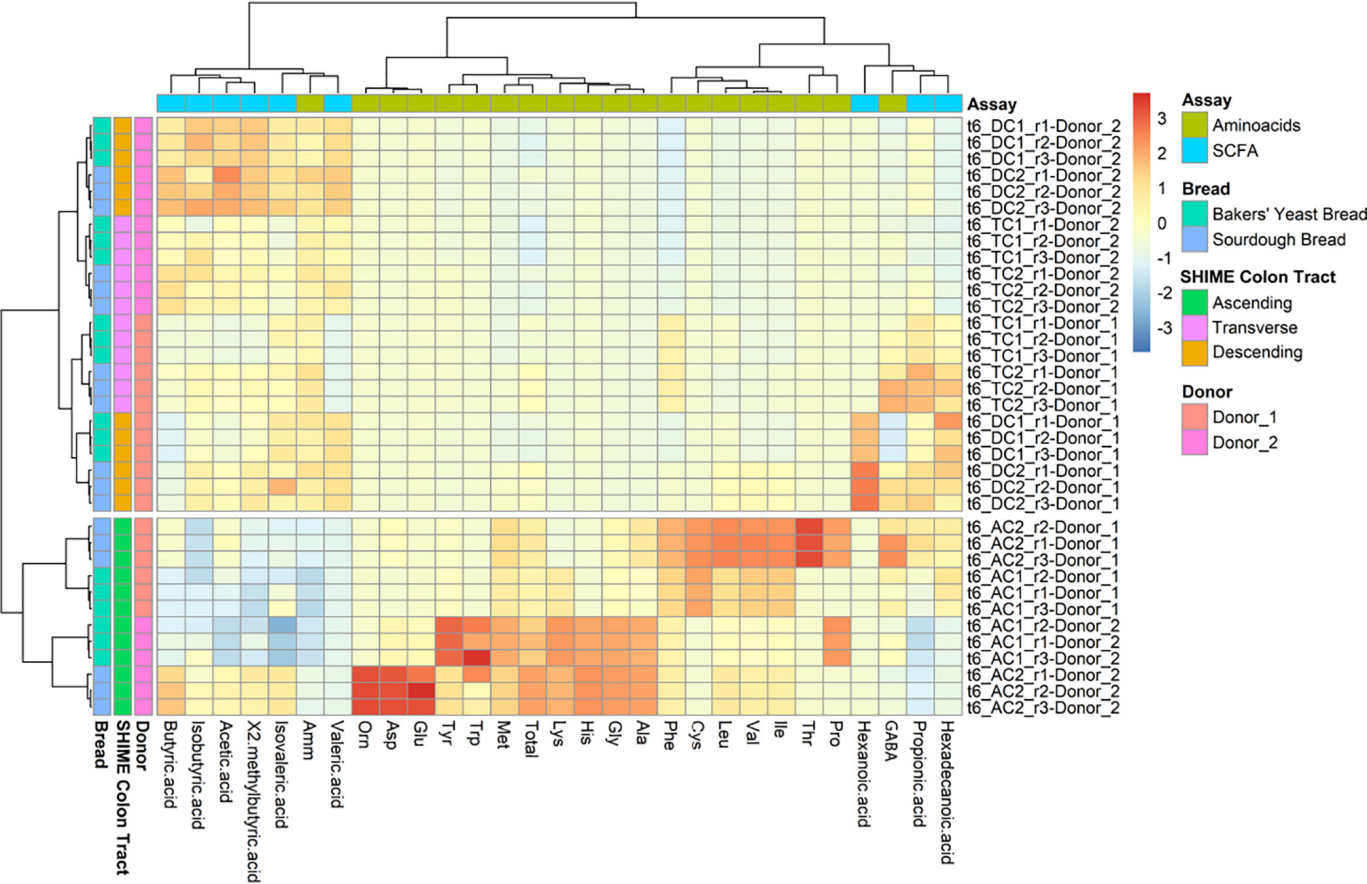
Pseudo-heatmap showing the clustering of free amino acids (FAA) and short chain fatty acids (SCFA) and their derivatives for donors 1 and 2, Twin M-SHIME colon tracts (ascending, transverse, and descending mucosa and lumen compartments) and after 14 days of feeding with baker’s yeast (BYB) and sourdough.

Metabolites such as FAA (Asp, Thr, Glu, Gly, Ala, Cys, Val, Met, Ile, Leu, Tyr, Phe, GABA, Amm, Orn, Lys, His, Trp, and Pro), SCFA (acetic, propionic, isobutyric, butyric, isovaleric, 2-methylbutyric, valeric, hexanoic, and hexadecanoic acids), and volatile components (VOC) belonging to several chemical classes (aldehyds, alcohols, esters, ketones, furans, and acids) were monitored before and after 14 days of bread feeding. Initially, we used a correlation heatmap analysis to match the above metabolites with donors, Twin M-SHIME colon tracts (lumen compartments), and types of bread. The first data set demonstrated that VOC affected the variability of the plot only because of the variability between the donors. Therefore, we removed VOC from the definitive heatmap of Fig. 4. Two main clusters were observed depending on the Twin M-SHIME colon tract. Ascending colon formed a separate cluster. Inside this, the main sub-class was represented by donors and, in turn, grouping was according to BYB and t-SB30 breads. Feeding with t-SB30 bread markedly affected the content of FAA, mainly Orn, Asp, and Glu for donor 2, and Val, Ile, and Trp for donor 1. Almost the same trend was observed for the second cluster including transverse and descending colon. With variations for absolute values between donors, acetic, butyric, and propionic acids were found at the highest levels in all Twin M-SHIME colon tracts after feeding with t-SB30 bread. SCFA derivatives did not markedly affect the clustering, except for hexanoic and hexadecenoic acids, which were found at higher levels in the ascending colon of donor 1, especially after feeding with t-SB30 bread. With variations among the colon tracts, the content of GABA was higher after feeding donor 1 with t-SB30. Table S3 reports the absolute values of all FAA and SCFA, and their derivatives pooling the contents of both donors. Except for Orn, one-way ANOVA analysis demonstrated that the contents of all FAA were significantly higher (*P*  <  0.05) after bread feeding. The same was found for acetic, propionic, butyric, and isovaleric acids. We used a general linear regression analysis to distinguish the effect of the types of bread (Table 2, Fig. S10). Compared with BYB bread, an increase of the content of all SCFA (acetic, propionic, and butyric acids) and some of their derivatives (isovaleric and 2-methylbutyric acids) was significantly (*P*  <  0.05) evident after 14 days of feeding with t-SB30 bread. The content of Asp, Thr, Glu, GABA, ammonia, and Orn was also significatively (*P*  <  0.05) higher when feeding was with t-SB30 bread. The same trend was already observed when the analyses were carried out after 7 days of feeding. Following 1 week of wash out, the content of FAA and SCFA and their derivatives of all colon tracts (lumen compartments) of both donors trended to similar absolute values of those found before bread feeding (Table S3).

**TABLE 2:** Estimates and significance for bread factor (baker’s yeast, BYB, and sourdough, t-SB30 breads) from the general linear regression after 14 days of feeding of both donors and all Twin M-SHIME colon tracts (lumen compartments)

Compounds	Estimate	Std. error	*P*-value
Short chain fatty acids (SCFA)			
Acetic acid	20.350	2.133	<0.001*
Propionic acid	5.782	0.925	<0.001*
Butyric acid	11.254	0.855	<0.001*
Isobutyric acid	0.108	0.056	0.060
Isovaleric acid	0.180	0.048	<0.001*
2-methylbutyric acid	0.125	0.019	<0.001*
Valeric acid	0.164	0.170	0.343
Hexanoic acid	0.479	0.472	0.319
Hexadecanoic acid	0.006	0.015	0.656
Free amino acids (FAA)			
Asp	2.859	0.882	0.003*
Thr	0.269	0.072	<0.001*
Glu	3.387	1.125	0.005*
Gly	0.349	1.213	0.776
Ala	3.721	3.293	0.268
Cys	0.034	0.134	0.798
Val	3.559	1.565	0.031*
Met	1.032	1.531	0.506
Trp	−1.310	0.948	0.178
Pro	−0.008	0.058	0.894
Ile	2.485	1.103	0.032*
Leu	2.487	1.274	0.061
Tyr	−1.319	0.788	0.105
Phe	4.271	1.631	0.014*
GABA	1.463	0.239	<0.001*
Amm	13.957	5.316	0.014*
Orn	0.061	0.016	<0.001*
Lys	−0.065	0.040	0.114
His	0.357	1.303	0.786

*indicates significant differences (*P*  <  0.05).

## DISCUSSION

Acting as nourishment for both humans and gastrointestinal microbes, diet is an efficient tool to selectively alter the microbiota composition and, especially, its functionality (21). But mere descriptions of human dietary habits are only partly effective for this purpose, and approaches discriminating between food components are the only resolutive (4). *In vitro* research using gastrointestinal simulators is not only the complement to *in vivo* studies but becomes the most promising way to assess the effect of single food components because it *a priori* excludes interferences from other dietary factors and attenuates human physiology overlaps. In this study, we used the Twin M-SHIME as the worldwide scientifically validated gastrointestinal simulator (17, 18). Using gastrointestinal simulators, the accurate selection of fecal donors is the prerequisite to fill, at least in part, the gap with respect to *in vivo* studies. Initially, we recruited 40 individuals whose nutritional questionnaires and calculation of MDS demonstrated a consistent adherence to MD (22). The statistical elaboration of fecal microbiota and metabolome data highlighted that basic indexes, such as the ratio between Firmicutes and Bacteroidetes, and the concentration of SCFA and their derivatives, were those typical of individuals adhering to MD (20, 22). This allowed the selection of two highly representative fecal donors. After the Twin M-SHIME reached the steady state (4 weeks), the microbiota composition at phylum and family taxa was consistent with that of the fecal materials from both donors.

Bread is a worldwide staple food but its processing varies, mostly depending on the leavening agent, which affects the sensory, nutritional, and other healthy attributes (11). While making baker’s yeast bread is relatively easy based on well standardized protocols, 30 years of knowledge on sustainably sourdough fermentation highlighted that each sourdough is unique and its performance depends on microbial composition and technological parameters (11). Spreading the use of sourdough fermentation is a global challenge. Some *in vitro* and *in vivo* studies assessed the effect of sourdough breads on the mitigation of gastrointestinal diseases and only a few focused on gut microbiota alteration. Most of these studies did not deepen the sourdough characteristics but concluded that the physiological effects markedly varies depending on sourdough processing (23). In this study, we previously determined the microbiological, biochemical, structural, and nutritional features of baker’s yeast and sourdough breads. This characterization had multifaced values. It allowed the discrimination for attributes that were mainly responsible for different responses between breads, proved the typical features of our industrial processes, and outlined what unique characteristics a sourdough bread must have to better perform. The selected sourdough used in this study was already effective to enhance the bread *in vivo* digestibility (19).

Even using various diversity indexes, NMDS analysis did not show statistical differences for the phylum and family microbiota composition, neither comparing before and after bread feeding nor comparing between baker’s yeast and sourdough breads. This was observed for all three Twin M-SHIME colon tracts, lumen and mucosal compartments, and for both donors. We achieved almost the same conclusion investigating the genus core microbiota and merging data from all Twin M-SHIME colon tracts, and luminal and mucosal compartments. The few fluctuations of the relative abundances dealt with *Bifidobacterium* (mainly increased with sourdough bread), *Lachnoclostridium*, *Lachnospira*, *Biophila*, and Escherichia-shigella. Bacilli clade positively responded to both breads, and *Leuconostoc* and *Lactobacillus* varied according to feeding with sourdough and baker’s yeast breads, respectively. Our conclusion that daily sourdough bread consumption does not have a substantial effect on the microbiota composition almost agreed with the few literature data. An *in vivo* randomized cross-over study did not show significant differences of the fecal microbiota composition comparing the consumption (1 week) of 145 g wholegrain wheat sourdough bread and 110 g white wheat bread, with the microbiota remaining resilient throughout the bread intervention (16). Almost the same was found using batch cultures with feces from three IBS and three healthy donors, where the sourdough bread mainly favored the increase of *Bifidobacterium* compared with nonfermented bread (24). Feeding rats with sourdough bread, the main differences did not concern the microbiota composition but the expression of various protein families (15). In another study, which used fecal slurries to compare bread making processes, changes in the relative abundance of a few genera or families depended on the capability to breakdown complex carbohydrates (14).

SCFA are synthesized through colonic fermentation by complex processes involving diverse microbial species starting from the breakdown or conversion of mainly dietary fibers, but also proteins and peptides, into various end products (25, 26). Butyric acid is largely expended as energy by colonocytes, while propionic and acetic acids travel to the liver via the portal vein, with acetate that reaches peripheral tissues after entering the systemic circulation. SCFA and some of their derivatives regulate the metabolism of glucose and lipids, promote mineral absorption, stimulate proliferation and differentiation of intestinal enterocytes, reduce the prevalence of inflammatory diseases and antioxidative functions, and have a crucial role in the functions of the central nervous system (5). Compared with baker’s yeast bread, our study showed that the content of all SCFA and some of their derivatives markedly increased after feeding with sourdough bread. This emerged for all colon tracts and both the donors. Another study, using only fecal slurries, observed that sourdough bread led to high butyrate production supporting important butyrate-producers within the families *Lachnospiraceae* and *Ruminococcaceae* (14). The sourdough bread used in this study had an initial content of resistant starch ca. 65% higher than baker’s yeast bread, and almost the same differences were found in the lumen compartment of the Twin M-SHIME colon tracts. Although not fully elucidated, such difference relies on acidification and proteolysis occurring during sourdough fermentation, which affect the bread rheology and microstructure and, in turn, the starch sensitivity to human digestive enzymes (27). As a common feature for dietary fibers, resistant or nondigestible starch withstands degradation in earlier sections of the gastrointestinal tract and reaches unaltered the distal tracts of the colon (2). Here, resistant starch not only affects the glucose and lipid metabolism by regulating nutrient absorption (28), but also represents the favorite substrate for the microbial synthesis of SCFA (2). Although the content of resistant starch reaching the large intestine mainly affects the synthesis of SCFA, the food shape and microstructure also interfere (29). Compared with baker’s yeast bread, the industrial sourdough bread showed differences concerning specific volume, hardness, and fracturability. Inhomogeneity with discontinuous protein-rich areas dominated by starch granules were the main distinguishing traits of the sourdough bread microstructure. Typically, ca. 10% of the ingested proteins reach the large intestine (30) and undergo proteolysis by colonic microbiota yielding to peptides and FAA (31). Unlike enterocytes, colonocytes do not absorb FAA to a significant extent, which remain available for colon resident bacteria to a wide variety of metabolic end products (32). These metabolites are the most disparate, including SCFA and their derivatives, and compounds with potential neuroactive activity such as GABA (2, 32). As the proteolysis only occurs at a significant extent during sourdough fermentation (11), the sourdough bread used in this study had a content of peptides four times higher with respect of baker`s yeast bread. The difference for FAA was much more evident. As previously shown (19), this reservoir of FAA already present in the sourdough bread had an *in vivo* effect on bread digestibility and increased the absorption at blood plasma level (19). In addition, it may have also contributed to synthesis of SCFA. Furthermore, the abundant supply of peptides was probably hydrolyzed in all Twin M-SHIME colon tracts. Indeed, the accumulation of Asp, Thr, Glu, and Orn mainly distinguished the feeding with sourdough bread. Except for Glu, these FAA did not correspond to those found at the highest concentrations in sourdough bread, which demonstrated the *ex-novo* liberation at the level of Twin M-SHIME colon tracts. Feeding the Twin M-SHIME with sourdough bread also promoted a significant increase of GABA. Bacteria usually responds to stressful conditions, in particular acidity, activating the glutamate decarboxylase, which, at expenses of glutamic acid and protons, leads to the synthesis of GABA (33). This fully agreed with the relevant concentration of Glu, which accumulated in the Twin M-SHIME colon tracts as the consequence of feeding with sourdough bread. GABA, a four-carbon nonprotein amino acid, acts as the major inhibitory neurotransmitter of the central nervous system and is associated to the induction of antihypertensive, diuretic and tranquilizer effects, and diabetes prevention (34).

We believed to have used the most efficient approach to investigate the potential of different bread making processes on gut microbiota composition and functionality. The selection of fecal donors after a comprehensive screening of individuals adhering to MD and the use of the most scientifically validated gastrointestinal simulator allowed certain conclusions. Bread consumption and types of bread do not have the capability to alter the Twin M-SHIME colon microbiota composition. Most importantly, the consumption of sourdough bread has the potential to enhance the synthesis of SCFA and FAA at the colon level.

## MATERIALS AND METHODS

### Microorganisms.

Previously selected (19) Lactiplantibacillus plantarum CR1, Furfurilactobacillus rossiae CR5, and Saccharomyces cerevisiae E10 were inoculated into the wheat flour (Triticum aestivum cv. Appulo) dough used for sourdough preparation (35). Lactic acid bacteria were propagated for 24 h at 30°C on modified MRS broth (Oxoid, Basingstoke, Hampshire, United Kingdom), with the addition of fresh yeast extract (5%, vol/vol) and 28 mM maltose, at the final pH of 5.6 (mMRS). The propagation of S. cerevisiae E10 was at 30°C for 48 h on Sabouraud dextrose broth (Oxoid).

### Sourdough propagation.

Type I sourdough was made according to traditional protocol (19), which used a previously fermented dough with L. plantarum CR1, F. rossiae CR5, and S. cerevisiae E10. After the first fermentation, four back slopping steps were carried out, mixing 20% of the previously fermented dough with flour and water, and incubating for 8 h at 30°C. After four refreshments, the acidification rate and volume increase of the dough were stable, and the mature S was used for preparing the sourdough (S_24_) to be used in bread making. S_24_ was prepared after refreshing (mixing 20% of S with flour and water, DY of 160) at 30°C for 24 h.

### Sourdough characterization.

Presumptive lactic acid bacteria were enumerated on MRS (Oxoid) supplemented with cycloheximide (0.1 g/L) at 30°C for 48 h, under anaerobiosis (AnaeroGen and AnaeroJar, Oxoid). Yeasts were enumerated on Sabouraud dextrose agar (SDA) (Oxoid) medium supplemented with chloramphenicol (0.1 g/L) at 30°C for 48 h. Water/salt-soluble extracts (WSE) of sourdoughs were used to analyze peptides (36), FAA (37), and organic acids (34). FQ was the molar ratio between lactic and acetic acids. Fermentations were carried out in triplicate and each one was analyzed in duplicate.

### Bread Making.

Breads (14 kg for each type) were manufactured industrially. BYB was made mixing wheat flour (62.5% wt/wt), water (37.5% wt/wt), and 1.5% (wt/wt) of baker’s yeast. The fermentation lasted 2 h at 30°C. The t-SB30 consists of 30% (wt/wt) sourdough S_24_ (fermented for 24 h at 30°C, step I), which was mixed with flour (46.7% wt/wt) and water (23.3% wt/wt), and further incubated for 4 h at 30°C (step II) (19). The two breads were baked at 220°C for 30 min. Bread making was carried out in triplicate and each bread was analyzed twice.

### Bread characterization.

Protein (total nitrogen × 5.7), lipids, and ash were determined according to the AACC methods 46-11A, 30-10.01, and 08-01, respectively. Total carbohydrates were calculated as the difference (100 - [proteins + lipids + ash]). The determination of dietary fibers was carried out by the AOAC method 991.43. Instrumental Texture Profile Analysis (TPA) was carried out with a TVT-300XP Texture Analyzer, equipped with a cylinder probe P-Cy25S and the 3.8.0.5 software (TexVol Instruments, Viken, Sweden) (34). Specific volume was measured through the BVM-test system (TexVol Instruments). The bread crumb features were assessed after 24 h of storage using the image analysis technology with the UTHSCSA ImageTool (34).

IVPD of flours, sourdoughs, and breads was determined according to Akeson and Stahmann (1964) (38), with some modifications (39). Samples were subjected to a sequential enzyme treatment mimicking the *in vivo* gastrointestinal digestion and IVPD was the percentage of the total protein, which was solubilized after enzyme hydrolysis. The supernatant, which contained the digested proteins, was freeze-dried and used for further analyses. The digested protein fraction was completely hydrolyzed in 5.7 M HCl (1 mL/10 mg of proteins) at 110°C under nitrogen stream (39). FAA were determined in the hydrolysate (37), while tryptophan was estimated according to Pintér-Szakács and Molnán-Perl (1990) (40). Chemical score (CS), which estimates the amount of protein required to provide the minimal EAA pattern as redefined by FAO in 2007 (41), indicates the CS of the most limiting EAA that is present in the test protein. EAAI, BV, PER, and NI were calculated according to the models developed by Oser (1959) (42).

### Starch hydrolysis predicted glycemic index and resistant starch.

The starch hydrolysis assessment was performed using a procedure mimicking the *in vivo* digestion of starch (43). The wheat flour bread leavened only with BYB was used as the control to estimate the hydrolysis index (HI  =  100). The predicted GI of breads was calculated using the following equation: pGI  =  0.549 × HI  +  39.71 (44). Resistant starch was determined using the Megazyme Resistant Starch K-RSTAR kit (AACC Method 32–40.01).

### Bread microstructure.

Solutions of 1% and 0.1% (wt/vol) of fluorescein isothiocyanate (FITC) and Rhodamine B (RhoB, Sigma) in dimethylformamide were used for noncovalent labeling of starch and proteins, respectively. Slices (ca. 0.2 mm and 1 cm × 1 cm) of defrosted breads were observed using a Leica SP8LIA CLSM with 488 and 552 nm lasers. Fluorescence emission was between 503 and 540 (FITC) and 605–645 nm (RhoB). Images were captured with a 10× objective, and analyzed with the software LAS X (45).

### Selection of donors: recruitment, dietary information, and collection of fecal samples.

This study was approved by the Research Ethical Committee of the Free University of Bolzano (17.09.2020, registration number: Mi4FO Cod 2020_06). The recruitment was done with fully informed written consent from all volunteers. An initial cohort of 61 healthy volunteers (age between 19 and 50 years) was recruited (Table S4). Preliminarily, nutritional questionnaires dealing with dietary habits and food consumption frequencies were administered (46). Based on nutritional questionnaires, a MDS was calculated (22). Main food components and typical habits (eight indicators) of the MD, adjusted for sex, contributed to the determination of MDS, which ranged from 0 to 8 points (minimal to maximal adherence). The cutoff of 4 was used to assess a satisfactory adherence to MD (22). Twenty-one volunteers were excluded. Forty volunteers showing an MDS from 4 to 8 were recruited to collect fecal materials (10 g to 50 g per person) into sterile bags, added (ratio 1:2) of RNA later solution (Applied Biosystems, Foster City, CA), kept at 4°C. The mixture was homogenized into sterile bags using a stomacher apparatus (Stomacher 400 Circulator, Seward, United Kingdom). Homogenized samples were stored at −80°C until use.

### *In vitro* bread digestion.

*In vitro* bread digestion was according to Brodkorb et al. (47). The oral predigestion comprised food dilution 1:1 (wt/wt) with simulated salivary fluid and the exposure (2 min) of the diluted food to salivary amylase. Then, the oral bolus was diluted 1:1 (vol/vol) with simulated gastric fluid and gastric enzymes (pepsin and gastric lipase), and incubation lasted for 2 h at 37°C, using a pH gradient from 6.0 to 2.0. Finally, the gastric chyme was diluted 1:1 (vol/vol) with simulated intestinal fluid, bile salts, and pancreatic enzymes (pancreatin, trypsin, and chymotrypsin), and incubation at pH 7 was extended for further 3 h at 37°C under static dialysis with a membrane of 14 kDa.

### Twin-M-SHIME experiment.

The Twin M-SHIME (UGent/ProDigest) configuration operated with two parallel SHIME units, each one consisting of five consecutive bioreactors simulating stomach, small intestine, and ascending (AC), transverse (TC), and descending (DC) colon (48). Each colon bioreactor contained a mucosal layer, consisting of 60 mucin agar-covered microcosms placed into a polyethylene netting (49). Figure S11 illustrates the experimental design. Before starting, the Twin M-SHIME AC, TC and DC bioreactors were simultaneously inoculated with the same fecal sample from the same donor (50). The colonic microbiota was allowed for stabilization (2 weeks) and further steady state (2 weeks). During these 4 weeks, each colon bioreactor was supplied (three times per day) with 200 mL of predigested adult M-SHIME feed PDNM001B (ProDigest, Ghent, Belgium). Feeding of colon bioreactors during treatment was supplemented with 210 g/day (70 g, three times per day) of previously digested BYB or t-SB30 breads. According to dietary habits of many countries, the bread average daily dose is within 180 g–250 g (8). After bread feeding, 1 week of wash out was carried out. Before, during, and after bread feeding, lumen (20 mL) and mucosal (30 mucin agar-covered microcosms) samples were collected three times a week from each of the two units and each of the three colon bioreactors. Lumen samples were stored at −80°C until the analyses. Mucin agar-covered microcosms were washed three times with 0.1 M phosphate buffer solution (PBS, pH 7.0) to remove the luminal microbiota, and collected in 20 ml of PBS. A vortex treatment for 2 min detached sessile cells. The resulting cell suspension was centrifuged (10,000 rpm for 10 min) and the pellet was stored at −20°C until analyses. The same experimental design was repeated for each of the two donors.

### High throughput sequencing.

Fecal samples were subjected to total DNA extraction using the Spin Kit for Soil (MP Biomedicals, Italy). Primers targeting the 16S rRNA variable region V3-V4 (Escherichia coli position 341–805, forward 341F: CCTACGGGNGGCWGCAG and reverse 806R: GACTACNVGGGTWTCTAATCC) (51) were used for bacteria. Amplicons were cleaned using the Agencourt AMPure kit (Beckman coulter), and DNA was quantified using the Quant-iT PicoGreen dsDNA kit (Invitrogen). Quality and purity of the library was assessed with a high sensitivity DNA kit (Agilent, Palo Alto, CA, USA) by the Bioanalyzer 2100 (Agilent). Library preparation and pair-end sequencing were carried out at the Genomic Platform—Fondazione Edmund Mach (San Michele a/Adige, Trento, Italy) using the Illumina MiSeq system (Illumina, USA). Raw paired-end FASTQ files were demultiplexed using idemp (https://github.com/yhwu/idemp/blob/master/idemp.cpp) and imported into Quantitative Insights into Microbial Ecology (Qiime2, version 2018.2). Sequences were quality filtered, trimmed, de-noised, and merged using DADA2. Chimeric sequences were identified and removed via the consensus method in DADA2. A naive Bayes taxonomy classifier was trained on Silva database (52) through r132 reference sequences (clustered at 99% similarity) using q2-feature-classifier’s fit-classifier-naive-Bayes method, trimmed to the V3-V4 region of 16S rDNA and applied to paired-end sequence reads to generate an operational taxonomic unit (OTU) table.

### Short chain fatty acids analysis.

Fecal samples were extracted (20) and injected into a Trace GC Ultra gas chromatograph (Thermo Fisher Scientific, San Jose, CA, USA) coupled with a TSQ Quantum XLS tandem mass spectrometer (Thermo Fisher Scientific). A fused silica Stabilwax-DA column (30 m × 0.25 mm i.d. × 0.25 μm) (Restek Corporation, Bellefonte, USA) was used for the chromatographic separation. The MS detection operated on full-scan mode (EI at 70 eV, ion source temperature at 250°C, *m/z* values ranged from 40 to 300 Da and acquisition scan time 0.2 s) and multiple reaction monitoring acquisition mode (20). The GC-MS data processing was performed using the qualitative and quantitative software package XCALIBUR 2.2 (Thermo Fisher Scientific).

### Statistical analysis.

All statistical analyses were performed in R programming version 4.04. The selection of the two donors was based on the clustering of fecal microbiota abundances of the 40 recruited individuals, aggregated at family level, together with SCFA data. Clustering was done with the Manhattan distance matrix and ward.D2 method. Partial least-squares discriminant analysis (PLS-DA) was performed considering the adherence to MD as an independent variable and OTU abundances as feature for the model. Contribution of each feature was further explored and annotated with the explanatory independent variable level. Twin M-SHIME data clustering was also based on the Manhattan distance matrix. SHIME experiment was considered as a full factorial experimental design (2^2^·3^1^) to determine the effect of each factor (donor, Twin M-SHIME compartment, bread type) on each metabolite response (FAA and SCFA) and applying one-way ANOVA test correction. Data was fitted to the following generalized linear model:

$$y=\beta_{0}\;+\;\beta_{1}x_{1}\;+\;\beta_{2}x_{2}\;+\;\beta_{3}x_{3}\;+\;\beta_{12}x_{1}x_{2}\;+\;\beta_{13}x_{1}x_{3}\;+\;\beta_{23}x_{2}x_{3}\;+\;\beta_{12}x_{1}x_{2}\;+\;\beta_{123}x_{1}x_{2}x_{3}$$

Statistical analyses of microbiota data of both donors (in duplicate) was done using phyloseq and microbiome R packages (53). Alpha diversity (chao1, inverse Simpson, Gini Simpson, and Shannon indexes) was computed on relative abundances of each condition. Differences among bread feeding endpoint and before feeding were assessed through Kolmogorov-Smirnov test. Beta diversity was performed through NMDS by using Bray-Curtis distance. The core microbiota pseudo-heatmaps considered the taxa composition of all Twin M-SHIME colon tracts and lumen and mucosal parts per endpoint feeding compared with before bread feeding. Prevalence on generated pseudo‐heatmaps of the core microbiota corresponded to the frequency in which identified genus occurred in all three Twin M-SHIME colon tracts together with lumen and mucosal compartments using a detection threshold ranging from 0.1% to 9.1%. Bacterial taxa discriminating between endpoint bread feeding were assessed through LDA effect size (LEfSe) with default settings and using Twin M-SHIME colon tracts and compartments (lumen and mucosal) as class and subclasses, respectively (54). *P values* associated with microbial clades and feeding status identified by LEfSe were corrected for multiple hypothesis testing using Benjamini and Hochberg false discovery rate correction (FDR). Resulting corrected and uncorrected *P values* are reported in Table S5, as well as LDA scores.

### Ethics approval and consent to participate.

This study was approved by the Research Ethical Committee of the Free University of Bolzano (17.09.2020, registration number: Mi4FO Cod 2020_06).

### Data availability.

Data of sequences are fully available at the NCBI with the project number PRJNA733904 and ID SUB9745000.
