# Supplementary material for: Feeding with Sustainably Sourdough Bread Has the Potential to Promote the Healthy Microbiota Metabolism at the Colon Level
Source: Microbiol Spectr. 2021 Dec 1;9(3):e00494-21. doi: 10.1128/Spectrum.00494-21 (PMC8668080; doi:10.1128/Spectrum.00494-21)
Supplement: SUPPLEMENTAL FILE 1 — Supplemental material. Download SPECTRUM00494-21_Supp_1_seq8.pdf, PDF file, 1.6 MB [file spectrum00494-21_supp_1_seq8.pdf]

Table S1. Biochemical and microbiological characteristics of the sourdough (dough yield of 160) used for bread making. S, mature type-I sourdough (after four refreshments); S_{24}, sourdough produced by mixing S with wheat flour and water, and fermented at 30 °C for 24 h, respectively.

	S	S_{24}
pH	3.92 ± 0.02[a]	3.58 ± 0.02[b]
TTA Total titratable acidity (ml NaOH 0.1M/10g)	6.5 ± 0.1[b]	8.8 ± 0.2[a]
Lactic acid bacteria (Log cfu/g)	9.5 ± 0.1[b]	9.8 ± 0.1[a]
Yeasts (Log cfu/g)	7.3 ± 0.1[b]	7.5 ± 0.1[a]
Lactic acid (mmol/kg)	40.5 ± 0.2[b]	91.1 ± 0.2[a]
Acetic acid (mmol/kg)	9.8 ± 0.3[b]	20.5 ± 0.2[a]
Fermentation Quotient (FQ)	4.1 ± 0.2	4.4 ± 0.2
Total Free Amino Acids (g/kg)	0.80 ± 0.07[b]	6.77 ± 0.10[a]

[a-b]Values in the same row with different superscript letters differ significantly ($p < 0.05$) based on one-way ANOVA (Tuckey-Kramer). The data are the means of three independent analysis ± standard deviations (n =3).

Table S2. Proximal composition of the experimental breads. BYB; Baker's yeast bread made mixing wheat flour (62.5% w/w), water (37.5% w/w) and 1.5% (w/w) of baker's yeast and fermented for 2 h at 30 °C; t-SB30; sourdough bread made mixing 30% (w/w) of sourdough S_{24} (fermented for 24 h at 30°C, step I) with flour (46.7% w/w) and water (23.3% w/w) and fermented for 4 h at 30 °C (step II).

	**BYB**	**t-SB30**
Energy value (kj/100g)	1201 ± 52	1176 ± 51
Total carbohydrates (%, d.m.)	84.5 ± 0.2	83.5 ± 0.2
Dietary Fibre (%, d.m.)	3.1 ± 0.1	3.2 ± 0.1
Lipids (%, d.m.)	1.3 ± 0.1	1.2 ± 0.1
Proteins (%, d.m.)	10.9 ± 0.5	11.1 ± 0.4
Ash (%, d.m.)	0.5 ± 0.1	0.5 ± 0.1

$^{a-b}$Values in the same row with different superscript letters differ significantly ($p < 0.05$) based one-way ANOVA (Tuckey-Kramer). The data are the means of three independent analysis ± standard deviations (n =3).

Table S3. Overall (donor 1 + donor 2) content of free amino acids (FAA, ppm) and short chain fatty acids (SCFA) and their derivatives (mM) for all Twin M-SHIME colon tracts (lumen compartments) before and after feeding with bakers' yeast (BYB) and sourdough (t-SB30) breads and washing out period.

	AC				TC				DC			
	**Before treatment**	**After treatment BYB**	**After treatment t-SB30**	**After washing out**	**Before treatment**	**After treatment BYB**	**After treatment t-SB30**	**After washing out**	**Before treatment**	**After treatment BYB**	**After treatment t-SB30**	**After washing out**
**Amino acids**												
Asp	1.86 ± 0.09^{c}	8.08 ± 0.08^{b}	19.42 ± 0.18^{a}	1.99 ± 0.08^{c}	NF	NF	NF	NF	NF	NF	NF	NF
Thr	NF^{b}	NF^{b}	3.24 ± 0.26^{a}	NF^{b}	NF	NF	NF	NF	NF	NF	NF	NF
Glu	NF^{c}	7.35 ± 0.12^{b}	47.99 ± 1.04^{a}	NF^{c}	NF	NF	NF	NF	NF	NF	NF	NF
Gly	15.06 ± 0.56^{b}	44.77 ± 0.87^{a}	48.95 ± 1.15^{a}	16.31 ± 0.76^{b}	NF	NF	NF	NF	NF	NF	NF	NF
Ala	36.12 ± 0.87^{c}	109.54 ± 0.82^{b}	151.78 ± 0.98^{a}	37.41 ± 0.94^{c}	NF	NF	NF	NF	NF	NF	NF	NF
Cys	NF^{c}	4.04 ± 0.01^{b}	4.45 ± 0.09^{a}	NF^{c}	NF	NF	NF	NF	NF	NF	NF	NF
Val	9.56 ± 0.13^{c}	42.09 ± 0.32^{b}	62.24 ± 1.11^{a}	9.92 ± 0.77^{c}	NF^{c}	0.14 ± 0.01^{b}	0.17 ± 0.01^{a}	NF^{c}	0.37 ± 0.02^{a}	0.01 ± 0.01^{d}	0.11 ± 0.01^{c}	0.22 ± 0.07^{b}
Met	4.83 ± 0.08^{c}	69.26 ± 9.31^{a}	67.55 ± 6.76^{a}	8.23 ± 0.72^{b}	NF	NF	NF	NF	NF	NF	NF	NF
Ile	7.36 ± 0.13^{d}	33.48 ± 0.86^{b}	51.68 ± 0.95^{a}	12.11 ± 0.19^{c}	NF	NF	NF	NF	NF	NF	NF	NF
Leu	4.47 ± 0.05^{d}	34.87 ± 0.38^{b}	63.26 ± 1.61^{a}	7.27 ± 0.12^{c}	NF	NF	NF	NF	NF	NF	NF	NF
Tyr	4.79 ± 0.01^{d}	39.80 ± 7.22^{a}	20.60 ± 9.37^{b}	8.91 ± 0.21^{c}	0.03 ± 0.01^{c}	3.22 ± 0.09^{b}	6.26 ± 0.31^{a}	0.04 ± 0.02^{c}	0.01 ± 0.01^{c}	1.67 ± 0.04^{a}	1.57 ± 0.07^{a}	0.12 ± 0.09^{b}
Phe	12.38 ± 1.02^{d}	141.62 ± 6.48^{b}	165.13 ± 7.52^{a}	22.49 ± 2.42^{c}	1.91 ± 0.11^{c}	58.47 ± 6.11^{b}	67.99 ± 3.73^{a}	1.99 ± 0.13^{c}	2.23 ± 0.01^{c}	11.14 ± 0.03^{a}	11.56 ± 0.33^{a}	3.21 ± 0.01^{b}
GABA	NF^{c}	9.35 ± 0.75^{b}	13.66 ± 0.98^{a}	NF^{c}	NF^{c}	7.61 ± 0.21^{b}	12.44 ± 0.88^{a}	NF^{c}	NF^{c}	1.72 ± 0.13^{b}	2.24 ± 0.55^{a}	NF^{c}
Amm	148.64 ± 3.42^{d}	213.10 ± 7.69^{b}	295.90 ± 9.41^{a}	157.99 ± 4.11^{c}	199.12 ± 4.21^{d}	473.95 ± 6.11^{b}	507.84 ± 8.21^{a}	215.12 ± 4.21^{c}	214.15 ± 6.91^{d}	488.30 ± 9.29^{b}	527.76 ± 4.39^{a}	254.15 ± 6.91^{c}
Orn	2.22 ± 0.02^{a}	NF^{c}	0.73 ± 0.01^{b}	1.89 ± 0.12^{b}	NF	NF	NF	NF	NF	NF	NF	NF
Lys	0.99 ± 0.01^{b}	1.90 ± 0.05^{a}	1.11 ± 0.81^{ab}	1.26 ± 0.09^{b}	NF	NF	NF	NF	NF	NF	NF	NF
His	24.48 ± 0.08^{c}	39.15 ± 1.03^{b}	43.44 ± 0.56^{a}	25.22 ± 0.17^{c}	NF	NF	NF	NF	NF	NF	NF	NF
Trp	11.78 ± 0.13^{c}	29.38 ± 6.32^{a}	30.74 ± 9.21^{a}	14.35 ± 0.45^{b}	NF	NF	NF	NF	NF	NF	NF	NF
Pro	NF^{b}	1.89 ± 0.53^{a}	1.80 ± 0.53^{a}	NF^{b}	NF	NF	NF	NF	NF	NF	NF	NF
**SCFAs**												
Acetic acid	24.58 ± 1.56^{d}	92.32 ± 10.95^{b}	127.91 ± 10.44^{a}	39.18 ± 2.01^{c}	37.18 ± 1.45^{d}	102.33 ± 1.29^{b}	132.76 ± 3.99^{a}	44.94 ± 1.71^{c}	42.15 ± 1.44^{b}	112.49 ± 1.72^{b}	138.26 ± 3.77^{a}	57.12 ± 1.92^{c}
Propionic acid	20.17 ± 1.63^{c}	30.33 ± 2.93^{b}	52.50 ± 4.58^{a}	22.11 ± 1.42^{c}	24.66 ± 0.36^{d}	50.50 ± 0.30^{b}	65.92 ± 1.93^{a}	35.89 ± 0.45^{c}	24.70 ± 0.61^{d}	41.38 ± 0.67^{b}	58.90 ± 2.24^{a}	32.12 ± 0.92^{c}
Isobutyric acid	0.83 ± 0.03^{a}	0.74 ± 0.21^{ab}	0.52 ± 0.04^{b}	0.88 ± 0.02^{a}	1.08 ± 0.56^{c}	1.39 ± 0.04^{b}	1.86 ± 0.01^{a}	1.25 ± 0.47^{c}	1.35 ± 0.11^{c}	1.56 ± 0.04^{b}	2.10 ± 0.06^{a}	1.32 ± 0.23^{c}
Butyric acid	13.81 ± 0.07^{d}	44.54 ± 0.41^{b}	63.27 ± 1.01^{a}	19.16 ± 0.17^{c}	16.44 ± 0.59^{d}	57.53 ± 0.27^{b}	63.65 ± 1.74^{a}	23.41 ± 0.69^{c}	13.53 ± 1.42^{c}	45.31 ± 0.56^{b}	53.71 ± 1.60^{a}	13.91 ± 1.65^{c}
Isovaleric acid	1.47 ± 0.14^{b}	2.39 ± 0.41^{a}	2.20 ± 0.11^{a}	1.51 ± 0.12^{b}	1.52 ± 0.04^{b}	2.88 ± 0.24^{a}	2.85 ± 0.10^{a}	1.58 ± 0.06^{b}	1.87 ± 0.17^{b}	3.24 ± 0.08^{a}	3.29 ± 0.51^{a}	1.89 ± 0.17^{b}
2-methylbutyric	0.57 ± 0.02^{b}	0.63 ± 0.06^{b}	0.82 ± 0.06^{a}	0.59 ± 0.03^{b}	0.69 ± 0.10^{c}	0.98 ± 0.04^{b}	1.24 ± 0.02^{a}	0.79 ± 0.15^{c}	0.86 ± 0.08^{c}	1.16 ± 0.05^{b}	1.45 ± 0.03^{a}	0.90 ± 0.12^{c}
Valeric acid	0.11 ± 0.01^{a}	0.12 ± 0.02^{a}	0.13 ± 0.02^{a}	0.12 ± 0.02^{a}	0.15 ± 0.01^{b}	0.29 ± 0.01^{a}	0.29 ± 0.01^{a}	0.17 ± 0.01^{b}	4.13 ± 0.34^{b}	5.63 ± 0.18^{a}	5.62 ± 0.16^{a}	4.21 ± 0.46^{b}
Hexanoic acid	0.03 ± 0.01^{a}	0.05 ± 0.01^{a}	0.03 ± 0.02^{a}	0.04 ± 0.02^{a}	0.01 ± 0.01^{b}	0.04 ± 0.02^{a}	0.03 ± 0.02^{a}	0.02 ± 0.02^{b}	2.37 ± 0.26^{a}	0.10 ± 0.01^{c}	0.70 ± 0.01^{b}	2.29 ± 0.29^{a}
Hexadecanoic	0.35 ± 0.11^{a}	0.46 ± 0.08^{a}	0.42 ± 0.04^{a}	0.37 ± 0.12^{a}	0.35 ± 0.14^{b}	0.33 ± 0.03^{b}	0.56 ± 0.07^{a}	0.36 ± 0.12^{b}	0.28 ± 0.03^{b}	0.66 ± 0.13^{a}	0.51 ± 0.12^{a}	0.30 ± 0.06^{b}

[a-d]Values in the same row with different superscript letters differ significantly ($p < 0.05$) based one-way ANOVA (Tuckey-Kramer). The data are the means of three independent analysis ± standard deviations ($n = 3$). NF means not found.

Table S4. Exclusion criteria for study eligibility

**Exclusion criteria**
Citizenship not belonging to countries of the Mediterranean basin
Omnivorous diet consolidated for less than 1 year, without exclusion of foods of the Mediterranean diet
Smoking or alcohol abuse
Age under 18 or over 50
Habitual use of medicines
Regular consumption of probiotics and prebiotics in the form of pharmaceutical preparations
Consumption of antibiotics in the 3 months prior to recruitment
Evidence of intestinal diseases (Crohn's disease, ulcerative colitis, manifest microbial dysbiosis, constipation, celiac disease, irritable bowel syndrome), and other conditions (type I or II diabetes, cardiovascular or cerebrovascular disease, cancer, neurodegenerative disease, rheumatoid arthritis, food allergies and intolerances)
State of pregnancy and lactation

Table S5. Resulting LDA scores, significant levels, and FDR-corrected p-values for identified community drivers before, and after feeding with bakers' yeast (BYB) and sourdough (t-SB30) breads for Donor 1 and Donor 2. Data are related to Figure 3.

Taxonomic Hierarchy	class	LDA.bt	p-value	q-value (FDR)
**Donor 1 Lefse LDA**				
Bacteria.Proteobacteria.Gammaproteobacteria	Bef. Feeding	3.66	0.00245	0.06604
Bacteria.Proteobacteria	Bef. Feeding	3.78	0.00034	0.01531
Bacteria.Firmicutes.Bacilli.Lactobacillales.Lactobacillaceae.Lactobacillus	BYB	3.84	0.00040	0.01531
Bacteria.Proteobacteria.Gammaproteobacteria.Enterobacteriales	Bef. Feeding	3.65	0.00103	0.03084
Bacteria.Firmicutes.Bacilli.Lactobacillales	t-SB30	3.89	0.00027	0.01531
Bacteria.Firmicutes.Bacilli.Lactobacillales.Leuconostocaceae	t-SB30	3.90	0.00014	0.01531
Bacteria.Firmicutes.Bacilli.Lactobacillales.Lactobacillaceae	BYB	3.84	0.00040	0.01531
Bacteria.Firmicutes.Bacilli	t-SB30	3.89	0.00027	0.01531
Bacteria.Proteobacteria.Gammaproteobacteria.Enterobacteriales.Enterobacteriaceae	Bef. Feeding	3.65	0.00103	0.03084
Bacteria.Firmicutes.Bacilli.Lactobacillales.Leuconostocaceae.Leuconostoc	t-SB30	3.90	0.00014	0.01531
**Donor 2 Lefse LDA**				
Bacteria.Firmicutes.Clostridia.Clostridiales.Lachnospiraceae.Lachnoclostridium	Bef. Feeding	3.91	0.01825	0.23964
Bacteria.Firmicutes.Clostridia.Clostridiales.Lachnospiraceae.Blautia	Bef. Feeding	3.32	0.01864	0.23964
Bacteria.Firmicutes.Negativicutes.Selenomonadales.Veillonellaceae.Megasphaera	BYB	3.92	0.00889	0.12637
Bacteria.Proteobacteria.Gammaproteobacteria.Enterobacteriales.Enterobacteriaceae.Klebsiella	Bef. Feeding	3.35	0.00881	0.12637
Bacteria.Firmicutes.Clostridia.Clostridiales.Lachnospiraceae.Lachnospira	t-SB30	3.44	0.00393	0.06249
Bacteria.Proteobacteria.Gammaproteobacteria	Bef. Feeding	3.97	0.00247	0.04169
Bacteria.Proteobacteria.Gammaproteobacteria.Enterobacteriales	Bef. Feeding	3.96	0.00241	0.04169
Bacteria.Proteobacteria.Gammaproteobacteria.Enterobacteriales.Enterobacteriaceae	Bef. Feeding	3.96	0.00241	0.04169
Bacteria.Firmicutes.Clostridia.Clostridiales.Lachnospiraceae.Fusicatenibacter	t-SB30	3.18	0.00156	0.03250
Bacteria.Proteobacteria	Bef. Feeding	4.11	0.00017	0.00392
Bacteria.Proteobacteria.Deltaproteobacteria.Desulfovibrionales.Desulfovibrionaceae.Bilophila	Bef. Feeding	3.55	0.00017	0.00392
Bacteria.Firmicutes.Bacilli.Lactobacillales	BYB	4.12	0.00017	0.00392
Bacteria.Proteobacteria.Deltaproteobacteria.Desulfovibrionales.Desulfovibrionaceae	Bef. Feeding	3.55	0.00017	0.00392
Bacteria.Firmicutes.Bacilli	BYB	4.12	0.00017	0.00392
Bacteria.Proteobacteria.Deltaproteobacteria	Bef. Feeding	3.55	0.00017	0.00392
Bacteria.Proteobacteria.Deltaproteobacteria.Desulfovibrionales	Bef. Feeding	3.55	0.00017	0.00392
Bacteria.Firmicutes.Clostridia.Clostridiales.Lachnospiraceae.Anaerostipes	t-SB30	3.47	0.00006	0.00327
Bacteria.Firmicutes.Bacilli.Lactobacillales.Lactobacillaceae.Lactobacillus	BYB	3.95	0.00003	0.00211
Bacteria.Firmicutes.Bacilli.Lactobacillales.Leuconostocaceae	t-SB30	4.01	0.00002	0.00211
Bacteria.Firmicutes.Bacilli.Lactobacillales.Lactobacillaceae	BYB	3.95	0.00003	0.00211
Bacteria.Firmicutes.Bacilli.Lactobacillales.Leuconostocaceae.Leuconostoc	t-SB30	4.01	0.00002	0.00211

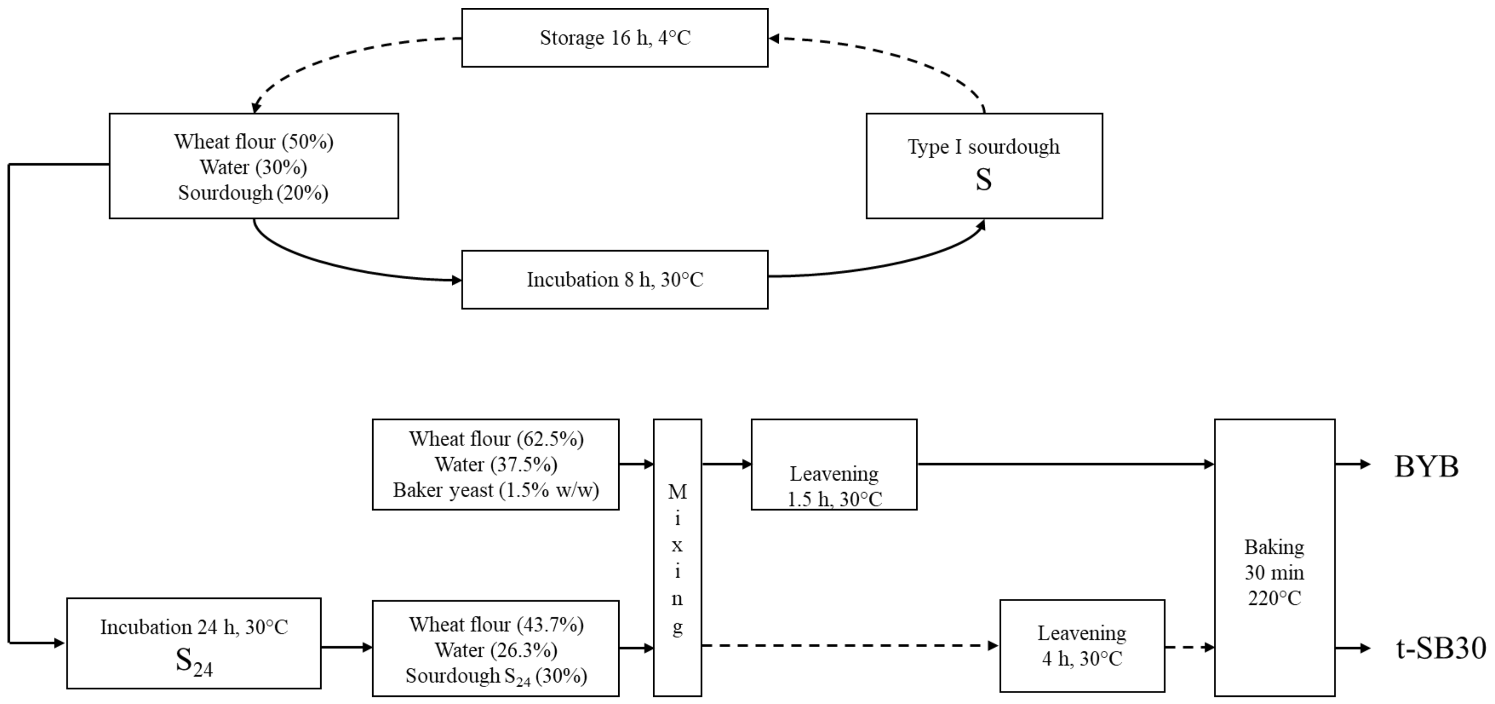

Figure S1. Workflow for bread making.

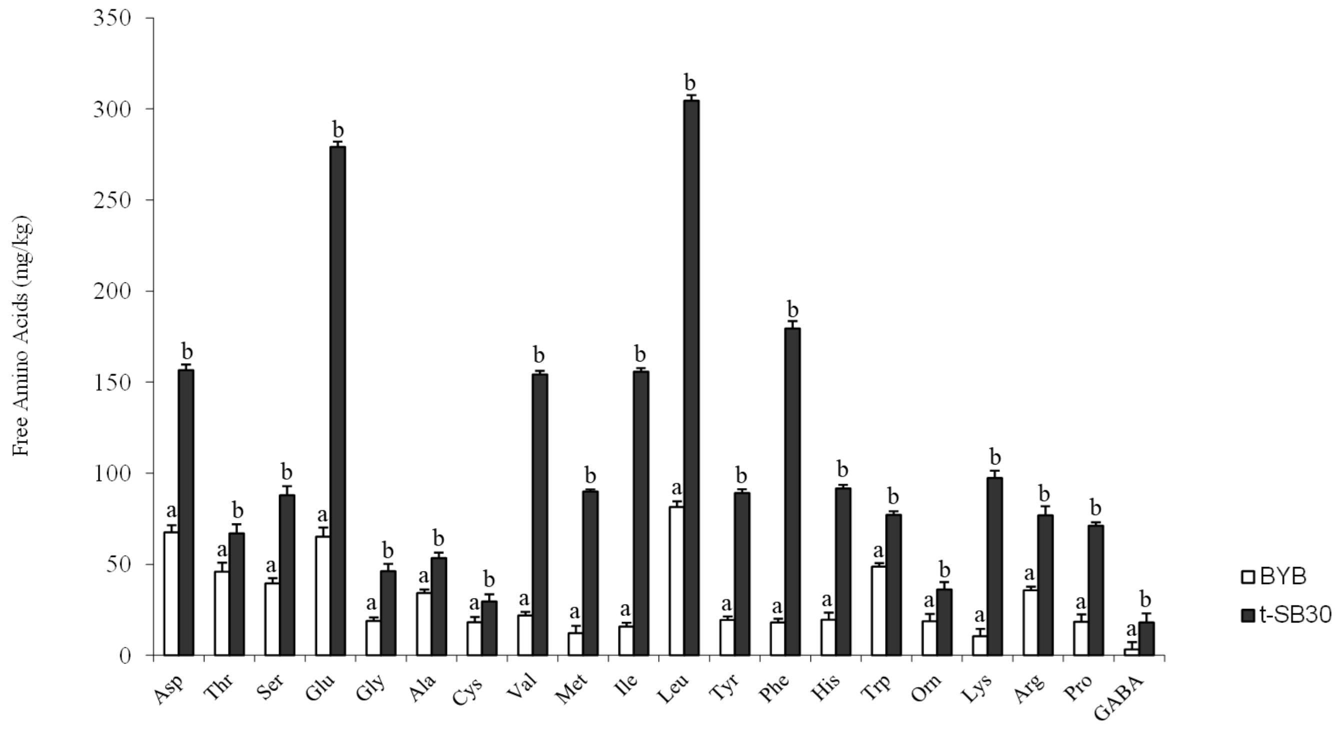

Figure S2. Concentration (mg/kg) of free amino acids (FAA) in experimental breads before baking. BYB; baker's yeast bread made mixing wheat flour (62.5% w/w), water (37.5% w/w) and 1.5% (w/w) of baker's yeast and fermented for 2 h at 30 °C; t-SB30; sourdough bread made mixing 30% (w/w) of sourdough S_{24} (fermented for 24 h at 30°C, step I) with flour (46.7% w/w) and water (23.3% w/w) and fermented for 4 h at 30 °C (step II). [a-b] FAA with different superscript letters differ significantly ($p < 0.05$) based on one-way ANOVA (Tuckey-Kramer). The statistical analysis was performed separately for each amino acid.

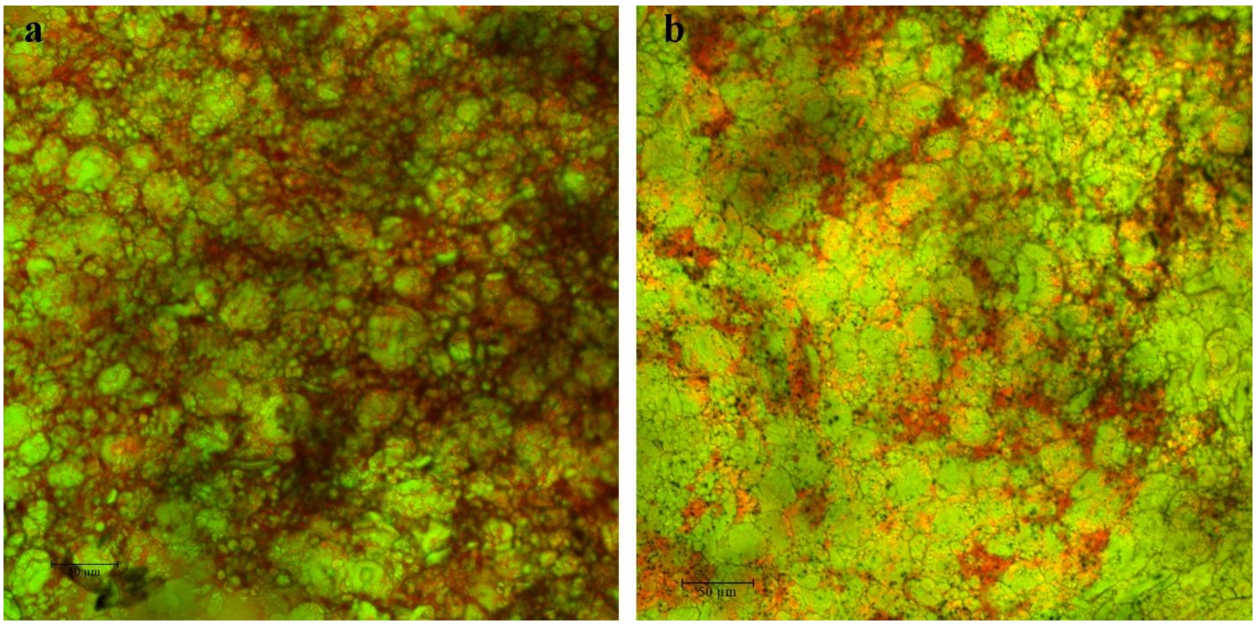

Figure S3. Confocal Laser Scanning Microscopy (CSLM) images of breads stained with fluorescein isothiocyanate (FITC) and Rhodamine B (RhoB). Green fluorescence: starch granules; red fluorescence: protein matrix. (a) Baker´s yeast bread (BYB); (b) sourdough bread (t-SB30). Bar = 50 µm.

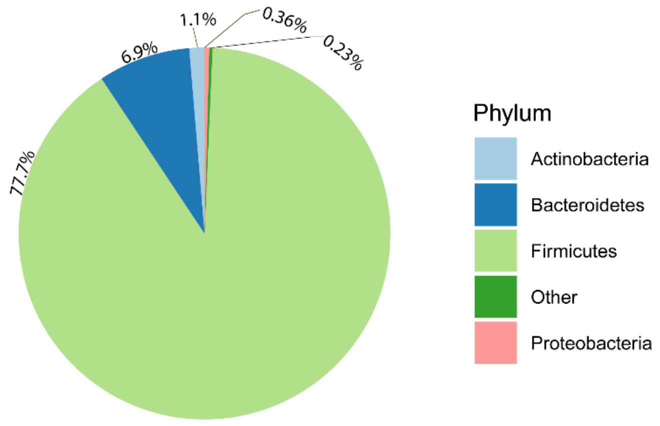

Figure S4. Aggregate microbiota composition (%) at phylum level for fecal samples for 40 recruited volunteers satisfactorily adhering to the Mediterranean diet (MD)**.**

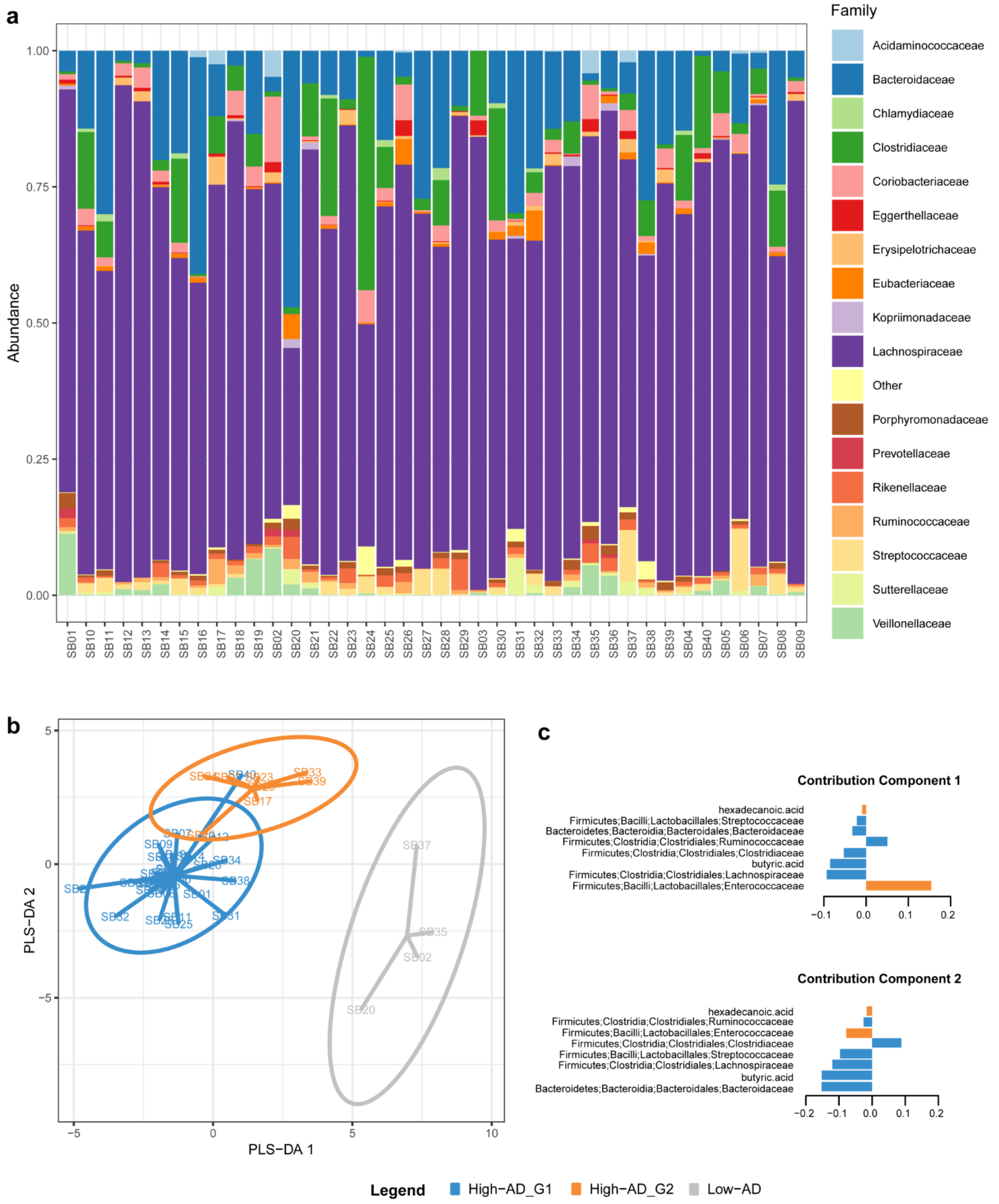

Figure S5. (a) Family relative abundance of the bacterial community of fecal samples from 40 recruited volunteers satisfactorily adhering to Mediterranean diet (MD). (b) Partial least square discriminant analysis (PLS-DA) of the three groups as shown by clustering of Figure S6 Blue and orange circles indicate, respectively, individuals belonging to groups 1 and 2, characterized by higher adherence to MD. Grey circle indicates group 3 characterized by less satisfactory adherence to MD. Components 1 and 2 describe 77.87% and 0.05% of variance, respectively. (c) Contribution of each variable to component loadings annotated by the most abundant group. Only components corresponding to groups with high adherence to MD are shown.

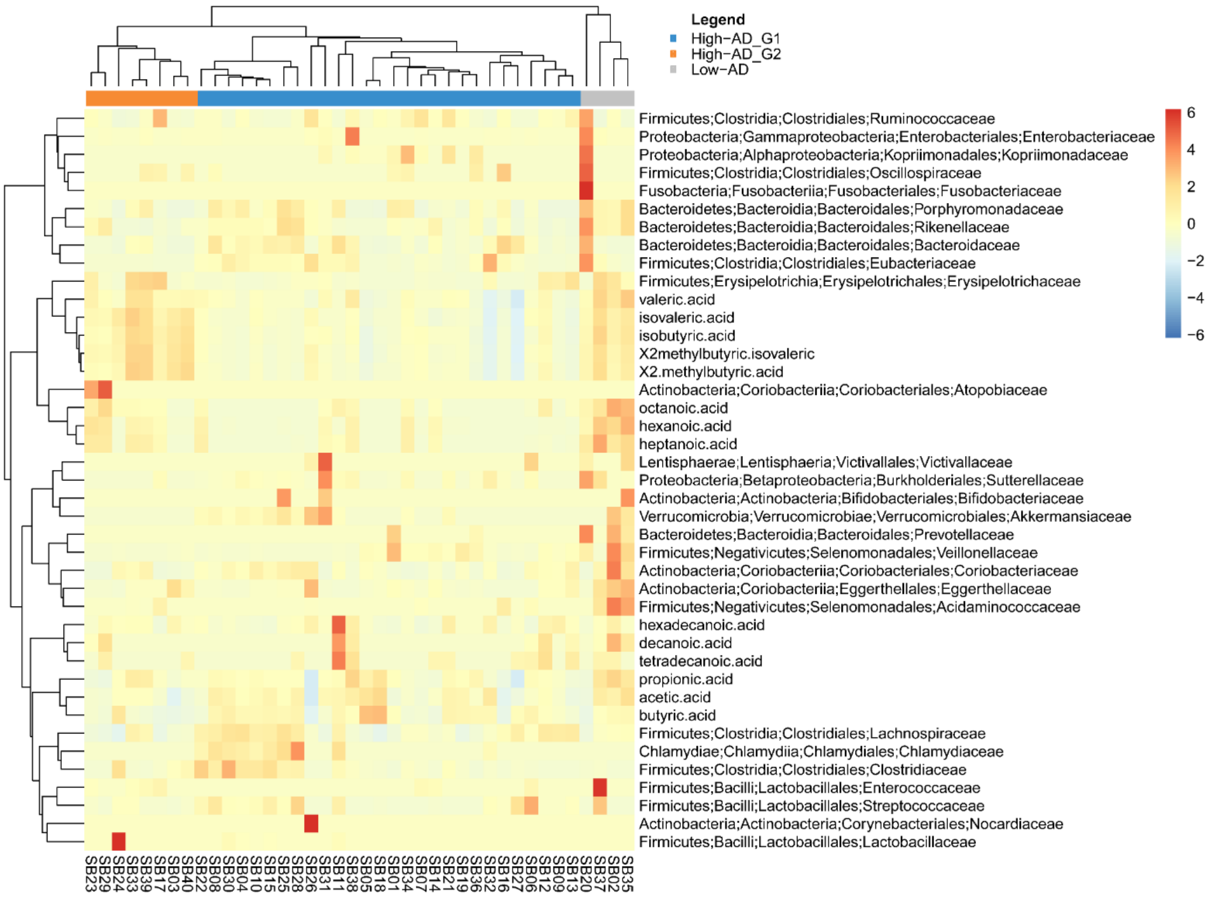

Figure S6. Clustering of 40 recruited volunteers according to fecal microbiota (family level) and metabolome (short chain fatty acids and their derivatives) composition. Blue (high adherence to Mediterranean diet – MD – group 1, High-AD_G1), orange (High-AD_G2) and grey (less satisfactory adherence to MD, Low-AD) indicate groups of individuals sharing similar microbiota and metabolome composition.

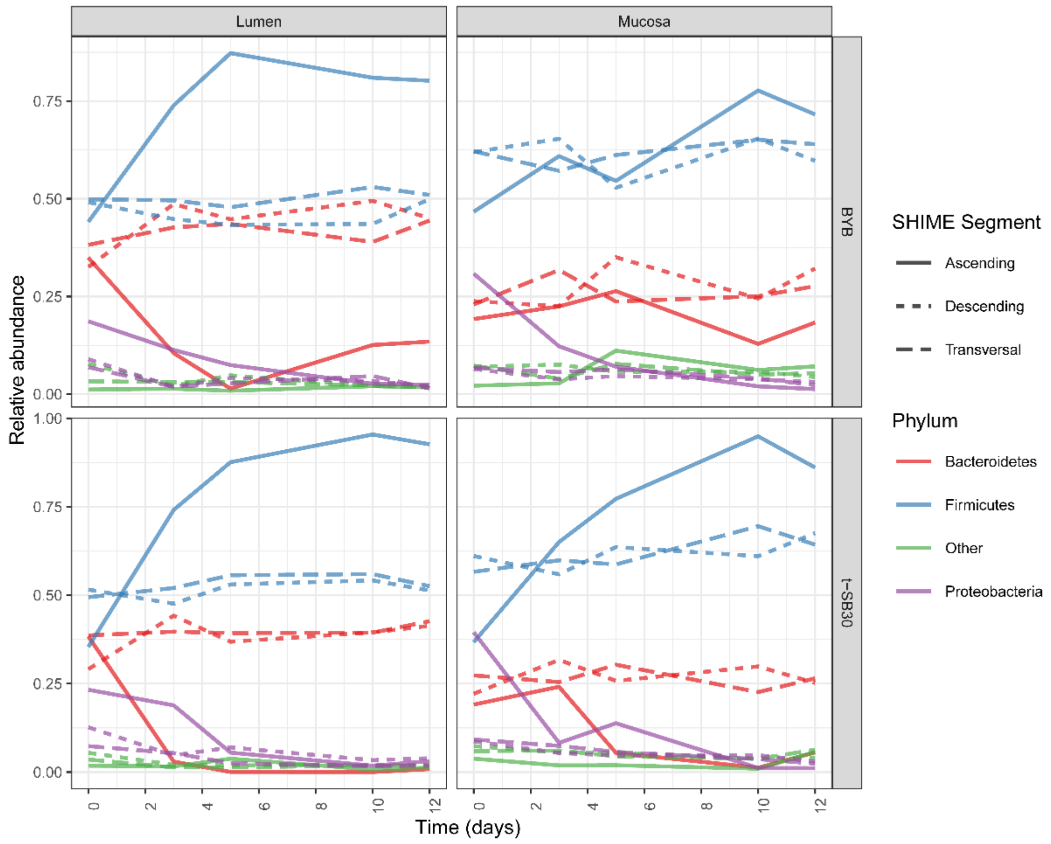

Figure S7. Microbiome variation over time at phylum level for donor 2 during the two-weeks of feeding with sourdough (t-SB30) and bakers' yeast (BYB) breads in both lumen and mucosal compartments.

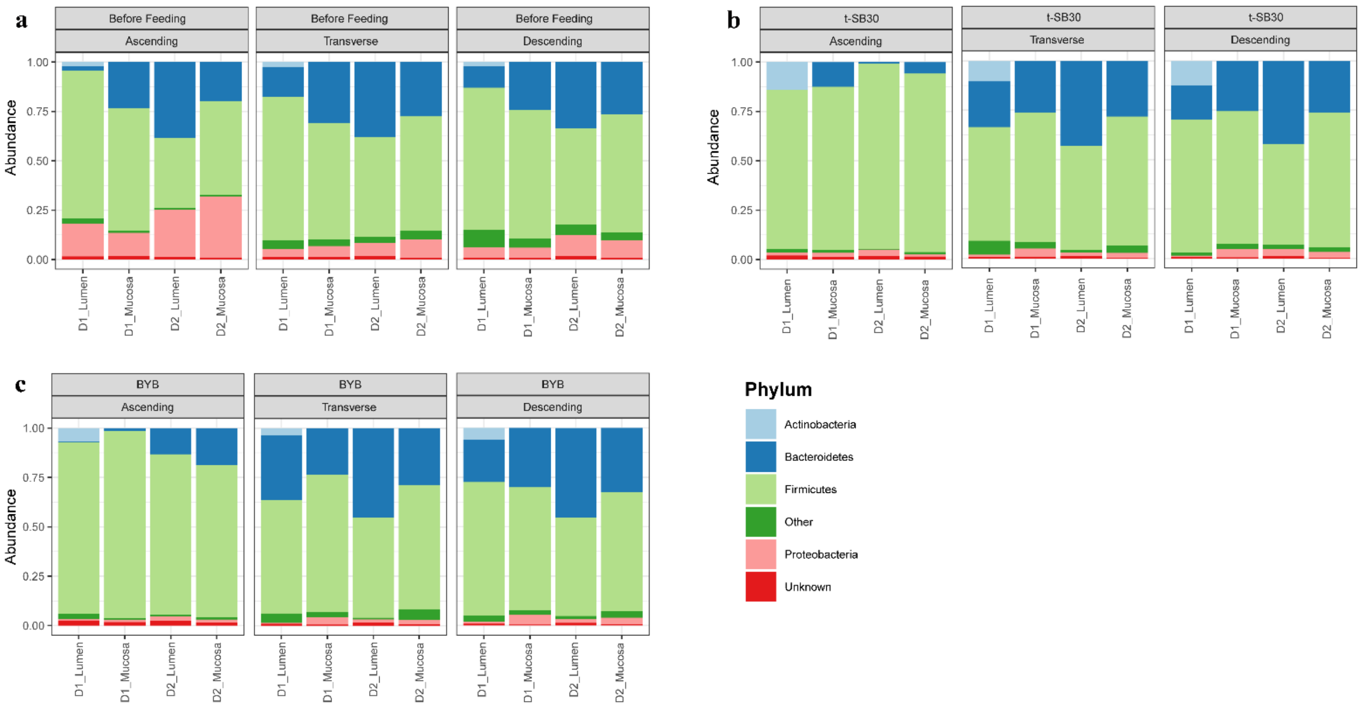

Figure S8. Relative abundance at phylum level of the microbiota, before (panel a) and after two weeks of feeding with sourdough (t-SB30) and bakers' yeast (BYB) breads (panel b and c, respectively), in the ascending, transverse and descending colon tracts (lumen and mucosal compartments) of Twin M-SHIME. t-SB30 (panel b) and BYB (panel c) indicate 14 days of feeding with sourdough and baker´s yeast breads, respectively. D1 and D2 prefixes indicate donors 1 and 2, respectively; Lumen and Mucosa suffixes indicate lumen and mucosal microbiota, respectively.

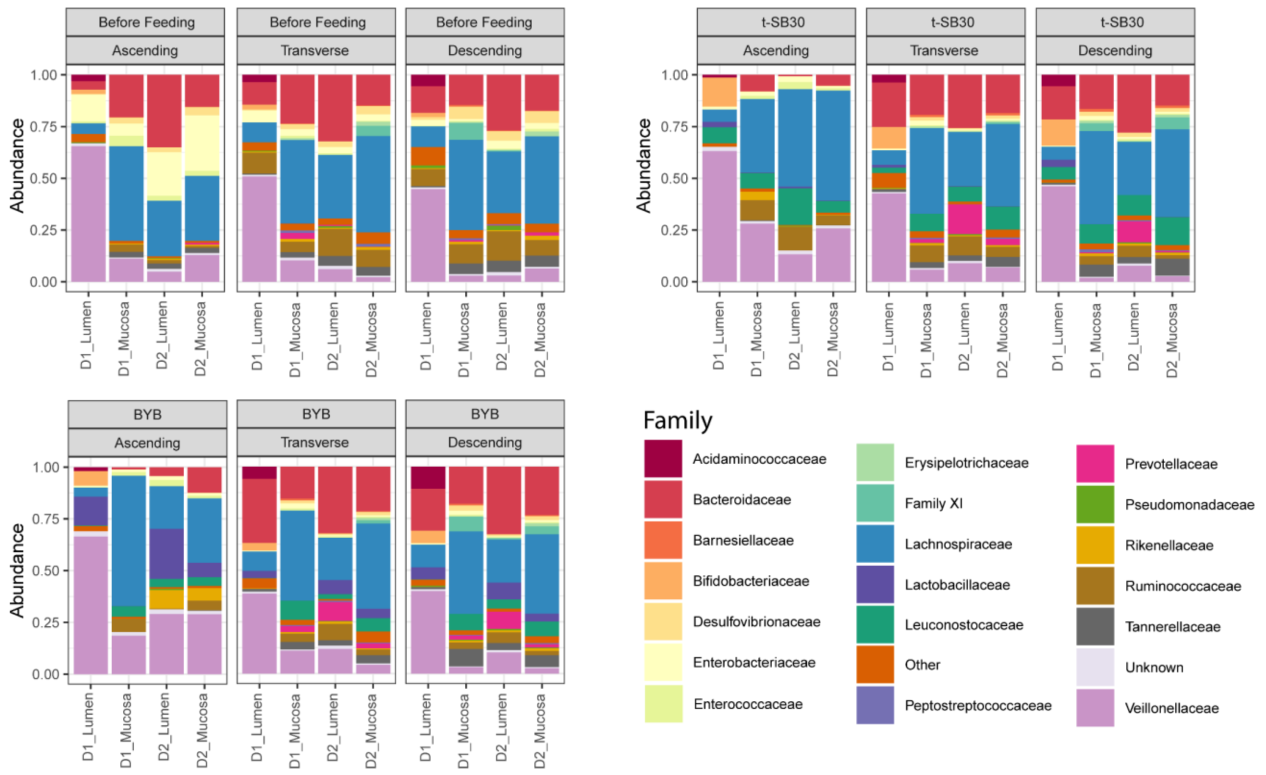

Figure S9. Relative abundance at family level of the microbiota, before and after two weeks of feeding with t-SD30 and BYB breads, in the ascending, transverse and descending colon tracts (lumen and mucosal compartments) of Twin M-SHIME. t-SB30 (upper-right graph) and BYB (lower-left graph) indicate 14 days of feeding with sourdough and baker´s yeast breads, respectively. D1 and D2 prefixes indicate donors 1 and 2, respectively; Lumen and Mucosa suffixes indicate luminal and mucosal microbiota, respectively.

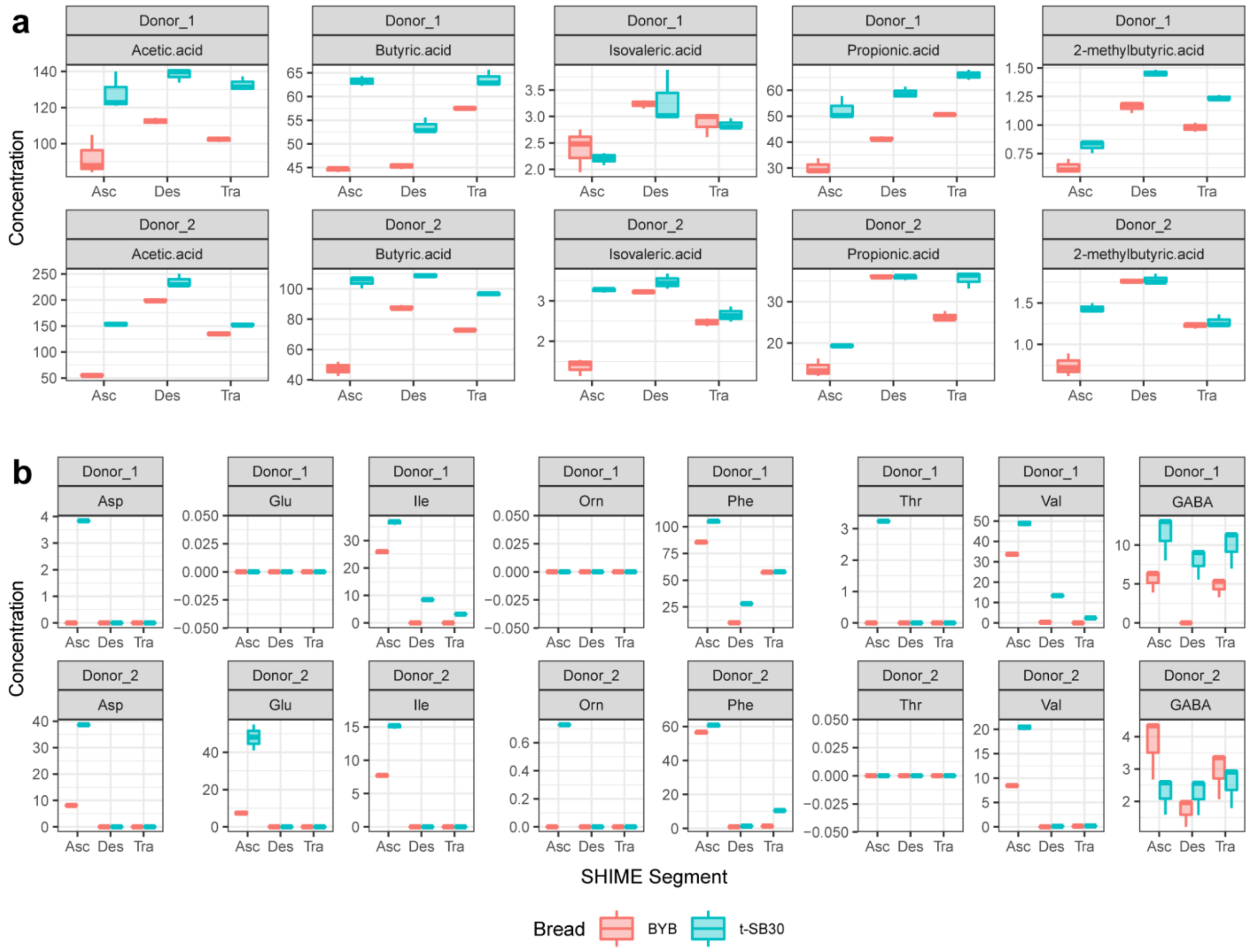

Figure S10. Boxplots of short chain fatty acid (SCFA) (panel a) and free amino acid (FAA) (panel b) after 14 days of feeding with bakers' yeast (BYB) and sourdough (t-SB30) breads of both donors in all Twin M-SHIME colon tracts (lumen compartments), expressed as mM and mg/Kg, respectively.

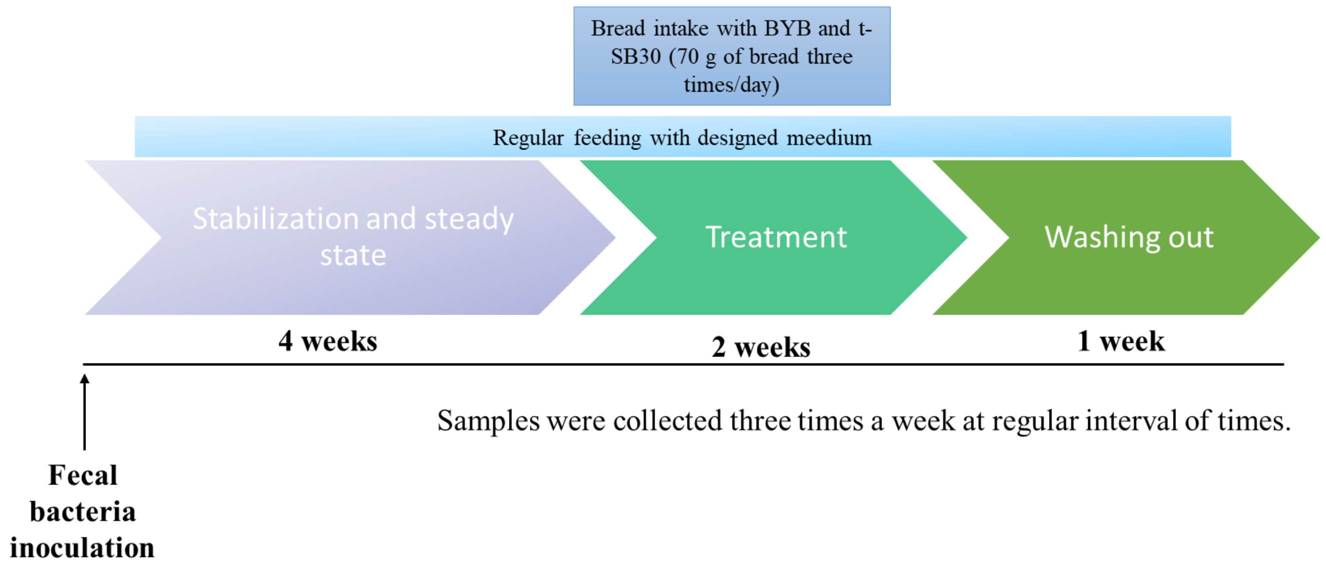

Figure S11. Experimental design of the Twin Mucosal-Simulator of the Human Intestinal Microbial Ecosystem (TWIN M-SHIME).
